# A possible origin of the inverted vertebrate retina revealed by physical modeling

**DOI:** 10.1007/s10867-024-09662-6

**Published:** 2024-08-03

**Authors:** Jan M. M. Oomens

**Affiliations:** Independent Researcher, Waalre, The Netherlands

**Keywords:** Eye evolution, Inverted retina, Eye prototype, Vertebrate eye, Spatial resolution

## Abstract

The evolutionary origin of the inverted retina in the vertebrate eye is unknown. This paper explores a hypothetical evolutionary scenario that explains the unique orientation of the photoreceptors in the vertebrate retina. The proposed scenario follows the scientific accepted scenario for eye evolution and gradually builds up towards an eye prototype by considering light direction detection and increase in achievable spatial resolution as the driving forces. It suggests that eye retinas developed along two different morphological processes, an evagination process that results in the inverted retina in vertebrate eyes and an invagination process that results in a verted retina in cephalopod eyes. The development of the inverted vertebrate retina and eye prototype morphology is strongly substantiated by physics of vision. The proposed evolutionary sequence for vertebrate eye development is simple and has the full potential to explain the origin of the inverted retina and leads to an eye prototype enabling visual detection and orientation. It allows the emergence of eye structures like, extraocular muscles, tapetum lucidum, biconvex lens, cornea, and pupil. This study supports the suggestion that a primitive inverted retina in the predecessor of vertebrates is of ectodermal origin and available before neurulation occurred.

**Supplementary Information:**

The online version contains supplementary material available at 10.1007/s10867-024-09662-6.

## Introduction

The eye is a highly complex organ comprising many components, and the direct evolutionary process that produces it has long eluded scientists. The evolutionary process of eye development began with light detection by sub-epithelial opsin expressing cells developing into the retinas of extant eyes. This identifies the retinas as the most ancient part of the eye, whereby two main types occur in the animal kingdom.

The first type of retina features light-facing photoreceptors (so-called verted retina) and is found in the eyes of flatworms, molluscs, and the camera eye of cephalopod molluscs [1], in contrast to the second type of retina in vertebrate camera eyes, in which the photoreceptor cells face away from the light (so-called inverted retina).

This article researches the possibility that this inverted character is an unavoidable result of a logically evolutionary morphological process.

Nilsson [2] and Land and Nilsson [1] extensively described the evolutionary development of the cephalopod mollusc camera-type eye. Nilsson [2] reported that the evolutionary process starts from a flat area of the skin, with light-facing photoreceptor cells that perceive local light levels. Through a cup-shaped invagination, this area develops directivity to light and detectable spatial resolution, enabling organisms to optically perceive their surroundings.

Detectable spatial resolution depends on three factors, the quality of the light receiving optics, the regularity and density of the photoreceptor matrix in the retina, and the signal processing capacity of the neural circuitry. It is assumed in this paper that all three aspects are balanced against each other.

For vertebrate camera-type eyes, it is unclear how the inverted retina emerges morphologically.

Lamb [3] proposed an evolutionary developmental trajectory of the vertebrate retina based on the current embryonic development of the photoreceptor cells and the vertebrate retina. That is after neurulation and does not explain the inverted retina. Furthermore, during evolutionary development, at some point at the macroscopic level, a spherical organ would emerge, with the axons running along the outside of the sphere. Although this is unlikely, it could explain how the axons end up in the optic stalk but it remains unclear why photoreceptors in the vertebrate retina face away from the light.

Carreras [4] proposed an evolutionary developmental pathway of the vertebrate retina based on a comparative study of embryogenesis of the cephalopod-type eye and the vertebrate eye involving morphogenesis, leading to the conclusion that the inverted retina was already available in the original ancestor of vertebrates before neurulation occurred. The proposed evolutionary developmental pathway does neither explain the inverted character of the inverted vertebrate retina.

In both studies, the origin of the inverted retinal characteristics remains unclear. At a macroscopic level, there is ample reason to explore a hypothetical evolutionary pathway that could explain the inverted orientation of photoreceptors in the retina of the predecessor of the vertebrate eye.

This hypothetical evolutionary pathway follows the evolutionary scenario for eye development presented by Nilsson [2] and is gradually built up by considering light direction detection and the increase in achievable spatial resolution as driving forces for eye prototype evolution. The evolution of the vertebrate retina and eye prototype morphology is strongly substantiated by physics calculations and should lead to an organ enabling accurate visual detection and orientation and allowing the emergence of eye components, such as the cornea, lens, and pupil. Combined with an increase in the number and regularity of photoreceptors in the retina, this leads to an improved spatial resolution, and thus a more detailed picture of one’s surroundings, improving the organism’s chances of survival.

## Method

The visual quality of the eye is determined by the achievable spatial resolution and thus by the density and regularity of the photoreceptor mosaic in the retina and the quality of the optics in the eye. Three mathematical models are developed in this study.

First, a geometric model was used to describe how the photoreceptor distribution changes during morphological development from a flat configuration to a spherical shape giving insight in the density and regularity of the photoreceptor matrix. Once the organ reached its spherical shape, a second model was developed to determine the image quality and size of the blurred spot on the retina. To assess the maximum detectable spatial frequency in light-sensitive organs, the theory of Nilsson and Pelger [5] was applied and modified to include the influence of the lens properties of a transparent spherical body.

The three models together provide good indicators of how the inverted retina and eye prototype may have originated from vertebrate predecessors.

**Fig. 1:**
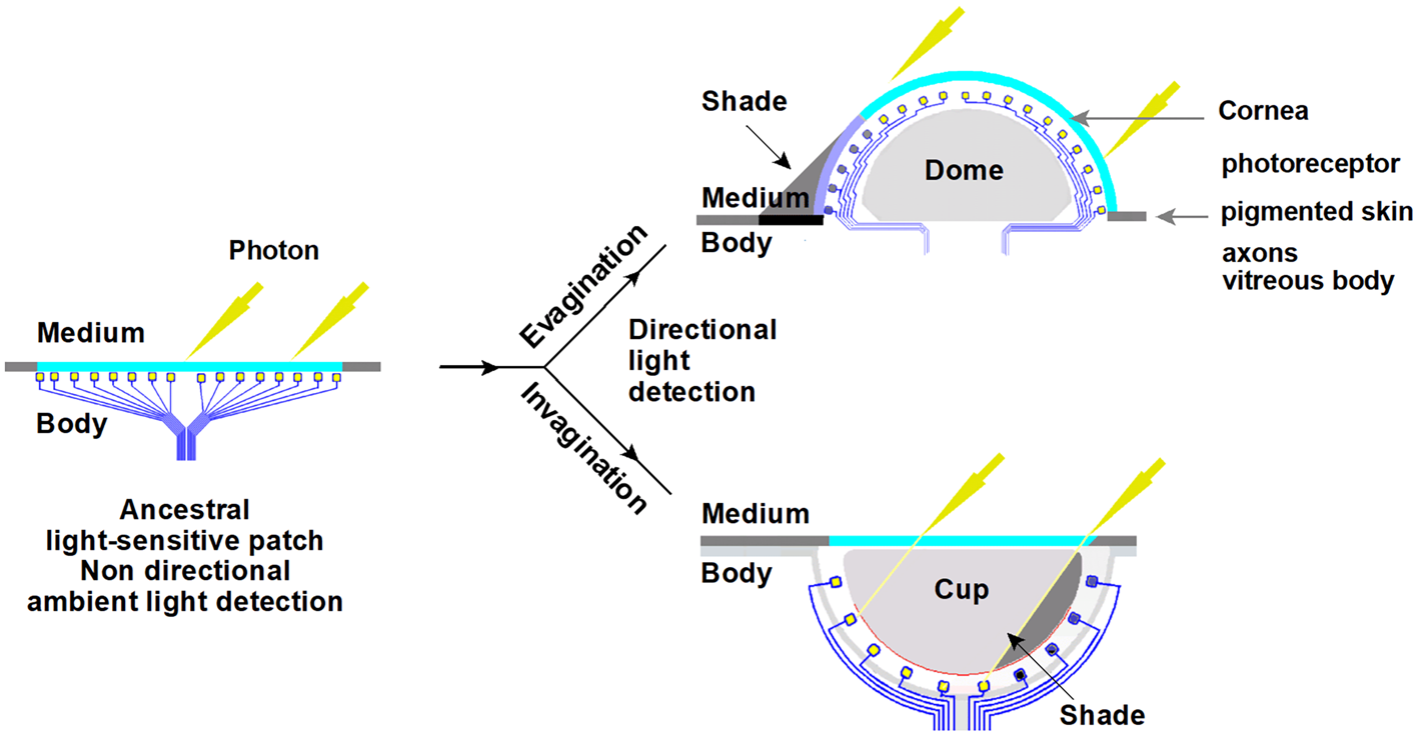
Cartoon of the development of a verted and inverted retina from a flat ancestral light-sensitive patch. The light-sensitive patch (**a**) is embedded in pigmented skin and features a transparent epithelial layer (turquoise) covering a layer of primitive photoreceptor cells (yellow box) directly connected with axons (blue lines) to the signal processing neural circuitry. The medium is water. It has two ways to create an organ receptive to the direction of light. Evagination (**b**) forming a dome supported by a vitreous body (gray) and invagination (**c**) forming a cup. Both configurations create a shaded space through refraction and shielding. Each photoreceptor in the patch can respond to a single photon and trigger a neural response. The positions of the axon pathways are considered to remain close to the light-sensitive surface. The axon pathways are omitted in further figures. Dome and cup configuration create directional light detection

According to [6], the vertebrate camera eye and the cephalopod camera eye share many structural similarities in morphological organization and are an example of convergent evolution. Both evolved independently from a common bilateral ancestor. About ~ 67% of genes expressed in both eye types trace back to this common bilateral ancestor [6], represented in Fig. 1 by the ancestral light-sensitive patch.

The ancestral light-sensitive patch in Fig. 1 contains widely spaced photoreceptors with axons, transparent supporting cells, and underlying capillaries. This study does not include retina development from a single layered to a multilayered retina. Color vision is not discussed.

The next step in the eye evolution scenario, directional light detection, can be easily achieved in two ways: invagination and evagination of a flat light-sensitive patch. Figure 1 provides a schematic overview of both the processes. The evagination branch (b) represents the development of the primitive inverted vertebrate retina. The invagination branch (c) represents the development of the verted retina in the cephalopod eye.

The development of the primitive vertebrate eye starts in the phylum Chordata possessing a pair of symmetrically positioned light-sensitive layers covered by a transparent epithelium. Basement membranes separate the layers. In the light-sensitive layer, photoreceptor cells are oriented towards the light. The parietal eye, as part of the epithalamus present in some vertebrates, is not discussed.

Albalat [7] concludes that the retinoid cycle machinery was not present in the ancestor of vertebrates and therefore only ciliary photoreceptor cells and ciliary (c)-opsins were present in the photoreceptor system of the ancestor of the vertebrates what means that there is no need to already consider RPE (retinal pigment epithelium) cells and photoreceptors together as a functional unit. Therefore, the flat light-sensitive photoreceptor patch (a) in Fig. 1 has no pigment cells because of the lack of function in this stage of ambient light detection. Pigmentation in the concave retinal layer gets its function when photoreceptors become more closely packed, and prevention of photoreceptor activation by stray light is needed to profit mostly from the increase of achievable spatial resolution. More information about the use of RPE cells and the shading effect of refraction is available in Online Resource 1.

The evagination scenario (b) in Fig. 1, in which a flat light-sensitive patch evaginates towards a spherical structure, has not yet been properly researched and, therefore, is explored in this study to find an explanation for the evolution of the inverted retina of the vertebrate eye. This research keeps the photoreceptors in the evagination scenario (b) at the surface of the bulging structure and the photoreceptors are ciliary cells using ciliary (c)-opsins capable of dim light detection.

As the light-sensitive area becomes more convex, it gradually covers an increasingly large field of view and allows the sensory nervous system to develop sensitivity to the direction of light. Finally, it develops into a light-sensitive sphere.

The opposite invagination scenario (c) in Fig. 1 is well described by Nilsson and Pelger [5] and Land and Nilsson [1] with respect to the evolutionary development of the cephalopod eye.

**Fig. 2:**
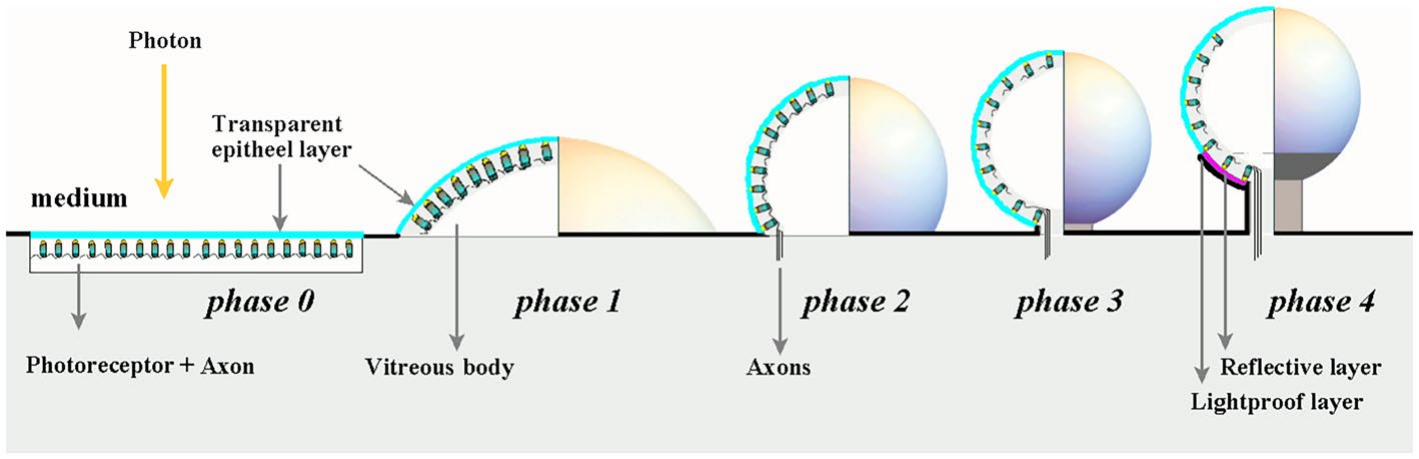
Cartoon showing the transformation of a flat circular light-sensitive patch into a spherical shaped organ on a stalk. The left half of every spere shows a cross section, the right half the outside. The photoreceptors in the patch are oriented to the light. The photoreceptor layer is supported by a transparent vitreous body when it protrudes into the medium, enlarging field of vision. The medium is water in phase 0–3 and water or air in phase 4. In the successive phases, the vitreous body gains refraction properties of a sphere. The phases follow the developmental steps proposed by Nilsson [2] and are as follows: 0. Patch of light-sensitive epithelia, containing ciliary photoreceptor cells using ciliary (c)-opsins, capable of detection of light during dim photic conditions allowing non-directional monitoring of ambient light. 1. Dome-shaped photoreceptor area, start of directional light sensitivity by refraction. 2. Sphere formation due to bulbous necking. Beginning of a posterior retina. 3. Posterior retina is fully developed and start of stalk growth. 4. Formation of an outer lightpro of layer, photoreceptor-free aperture, and on the inside of the outer lightproof layer, eventually a reflective layer to improve photodetection. Inverted retina activation and development of a primitive light-sensitive organ that allows visual tasks based on low spatial resolution

Figure 2 shows the phases of hypothetical evolutionary development of the light-sensitive sphere, where each phase introduces a new trait that improves an animal’s chances of survival.

The stalk extends the light-sensitive organ away from the body and magnifies the field of perception [8], while enabling ground-dwelling creatures to perceive prey and threats from above.

In phase 1, there was no spatial resolution or imaging possible, because of the convex curvature of the retina.

The first option to overcome this disadvantage, evolution let photoreceptors in the convex curved retina recess radially in individual ommatidia. Each ommatidium receives photons coming from a different portion of the outside world at a small solid angle, together, creating a compound eye with multiple optical units [1]. Dawkins mentioned this process in his book *Climbing Mount Improbable*. More detail is available in Online Resource 1.

A second option is to continue the bulging of the light-sensitive sphere on a stalk as presented in phases 2–3 to enlarge the field of view for bottom dwellers to hide their body in the seabed with their light-sensitive organs above the seabed. In that process, light direction detection in phase 2 and detectable spatial resolution in phase 3 arise as a “secondary effect” that triggered further development into a phase 4 eye prototype.

In phase 2, constriction and formation of a primitive light-sensitive spherical organ begins. Only the intensity and direction of light were observed.

In phase 3, the transparent light-sensitive organ can be understood as a fully developed sphere sparely covered by photoreceptors. A remarkable feature of the spherical, transparent, light-sensitive organ is that it behaves as a convex verted retina for the light directly falling on it, as well as a concave inverted retina for incident light.

In phase 4, an outer lightproof cell layer develops from the stalk into anterior direction blocking direct light and thereby restricting the retina underneath to act as a concave retina only. A concave curved retina allows for spatial resolution detection and primitive imaging. The two phase 4 symmetrical light-sensitive organs potentially enables the animal to perceive movement [1]. Surface dwellers can use the stalk eye to perceive above the water while their body remain under water.

Only photons entering the sphere anteriorly can activate the photoreceptors in the opposite posterior retinal region already covered with the lightproof outer layer. Vee et al. [9] has argued that a reflective layer (tapetum lucidum) evolved in vertebrates as a co-adaptation for better photodetection in the inverted retina in low-light conditions. It would already in an early stage increase the receptibility of the evolving light sensitive organ and as such the detectable spatial resolution under low-light conditions. This reflective layer is crystalline and can have different structures. One of the structures contains numerous rods arranged in a regular manner and stacked like masonry. This structure is reminiscent of the structure of the collagen fibrils in the stroma of the cornea. Morphologically, the covered transparent layer could have been transformed into the reflective tapetum lucidum without much structural change.

It is unlikely that photoreceptor coverage is entirely uniform across phases, as between phases 2 and 3, the photoreceptor density at the base of the sphere becomes particularly dense. To achieve an even photoreceptor distribution in the retina, significant photoreceptor migration in the support matrix should be necessary and, therefore, unlikely. An increase in photoreceptor cells through cell division will gradually fill in the vacant positions and lead to a more dense and more regular photoreceptor pattern in the retina.

It is reasonable to assume that photoreceptors retain their position within the support matrix of epithelial cells during the evagination process and that support matrix change is achieved by either the intermediate epithelial cells changing their shape or adapting the support structure to a spherical shape via cell division in the anterior part of the sphere and cell annihilation in the posterior part. The photoreceptor density distribution on the spherical organ is significantly different from the initial density distribution on the light-sensitive patch and has to be analyzed in a photoreceptor density model.

## The photoreceptor density model

**Fig. 3:**
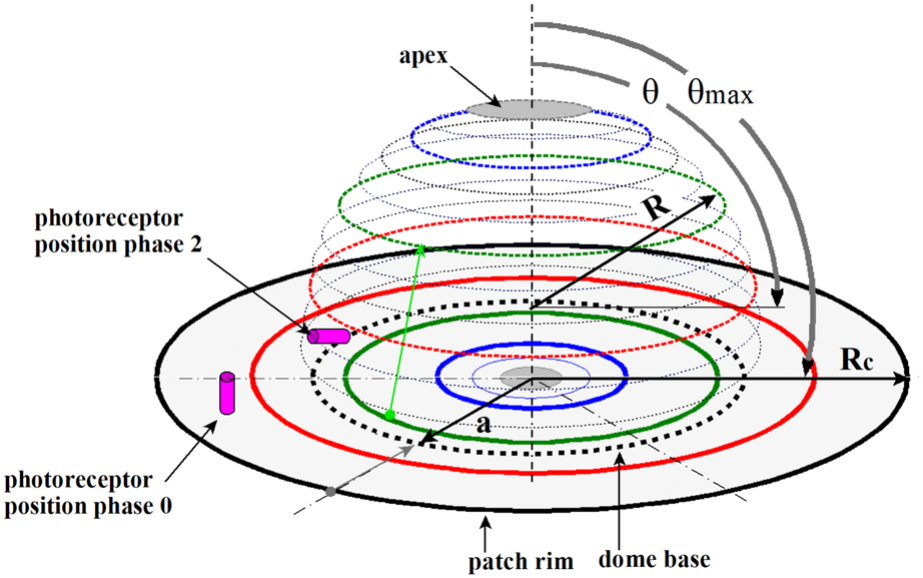
Schematic representation of the parameters involved in the transition of the flat circular light-sensitive patch to a hollow spherical cap. The flat circular patch (solid lines) represents phase 0 of Fig. 2. The dome (dotted lines) represents phase 2 of Fig. 2. The rim of the patch forms the base of the spherical cap. The patch (solid lines) has a fixed radius *R*_c_. The spherical cap (dotted lines) has a radius *R*, a cap base diameter of 2,∙and a spanned angle *θ*_max_ defined from the apex of the cap to its base. The polar angle *θ* = 90° marks the equatorial plane of the spherical cap. The colored rings schematically represent the initial and final positions of photoreceptors on that ring. Depending on the values of the three parameters, local tissue stretching and/or tissue shrinkage occurs. If the arc length over the dome *R*∙*θ*_max_ > *R*_c,_ the retina will stretch on a meridian and the corresponding distance between photoreceptors will increase proportional. Relative to the apex, the distances between photoreceptors on the parallels will continue to decrease from the apex to the base of the spherical cap. Together, these two outcomes alter the local photoreceptor density. The defined parameters are used in further mathematical models

In photoreceptor density modeling, a light-sensitive patch features photoreceptor cells that are evenly distributed over the surface. To calculate photoreceptor density distribution during morphogenesis, the initially flat surface was conceived as a plastically deformable membrane that rotationally symmetrical deforms into a light-sensitive sphere with a convex retina that protrudes into the medium (Fig. 3). The convex retina is supported by a transparent vitreous body that has the refractive properties of a lens, owing to its spherical shape. The number of photoreceptors is kept constant.

**Fig. 4:**
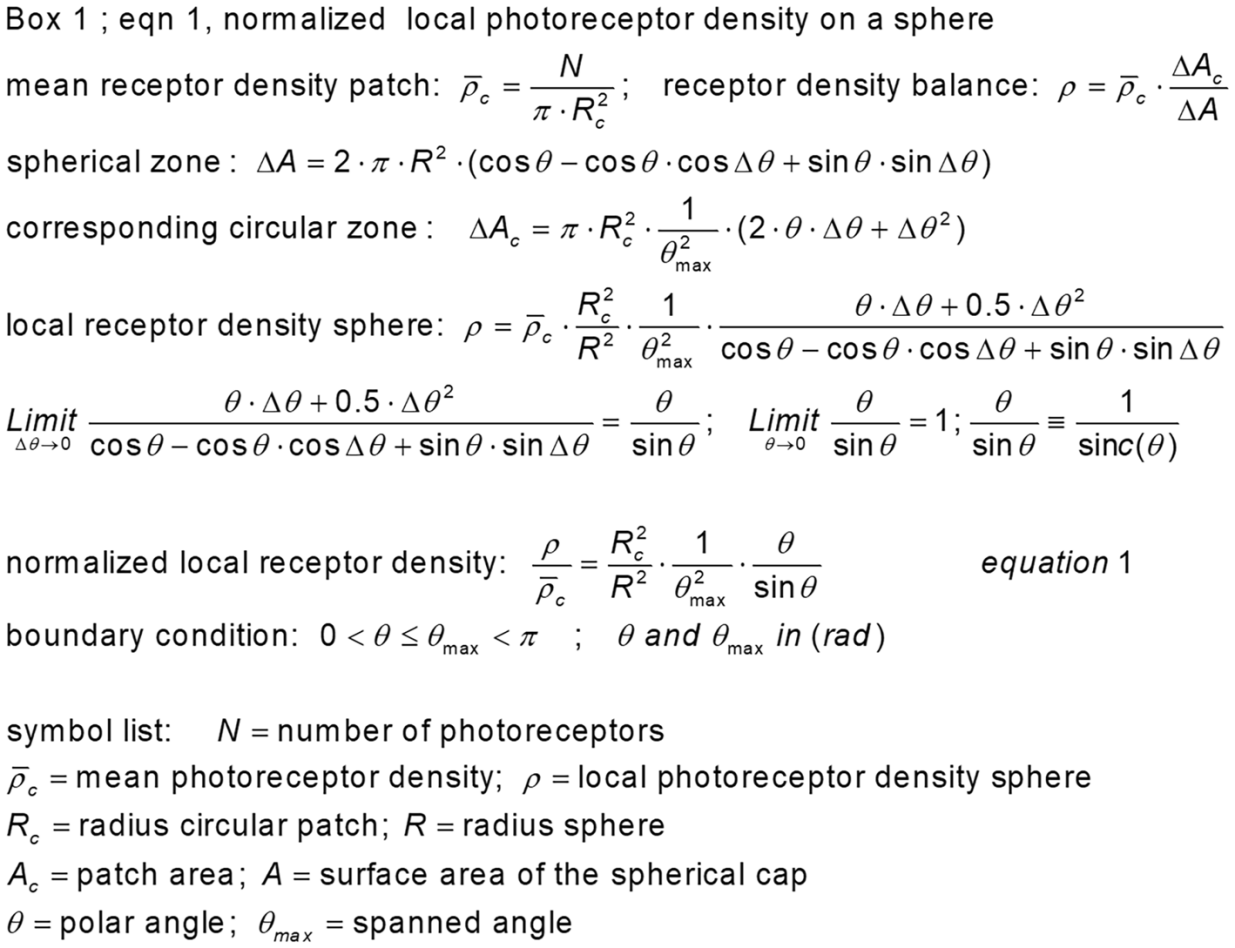
Normalized local photoreceptor density on a sphere. *R*_c_, *R*, and *θ*_max_ determine the final state of the evagination process and are therefore boundary conditions for Eq. (1) within which the local photoreceptor density can be calculated as a function of *θ* (radians). The circle radius *R*_c_ and $\overline{{\uprho }_{\text{c}}}$ are assumed to be constant and used as fixed reference values so that the local photoreceptor density on the sphere can be presented in a normalized manner. There is a relation between *R*_c_ and *R* that depends on the evagination scenarios that will be described in Fig. 5. A normalized local receptor density Eq. (1) where angles can be presented in degrees (°) is available in Online Resource 2

Figure 4 Box 1 states the derivation and equation for calculating the local normalized photoreceptor density on the surface of the developing spherical cap. The photoreceptor density distribution is a function of the circle radius *R*_*c*_, sphere radius *R*, angular position *θ*, and the spanned angle *θ*_max_ and is given in Eq. (1) (Fig. 4, Box 1).

Nilsson and Pelger [5] used a scenario in which the radius of the developing spherical cup was the same as that of the light-sensitive patch. This is the reason for analyzing the photoreceptor density on a developing spherical cap for the constant-radii scenario.

**Fig. 5:**
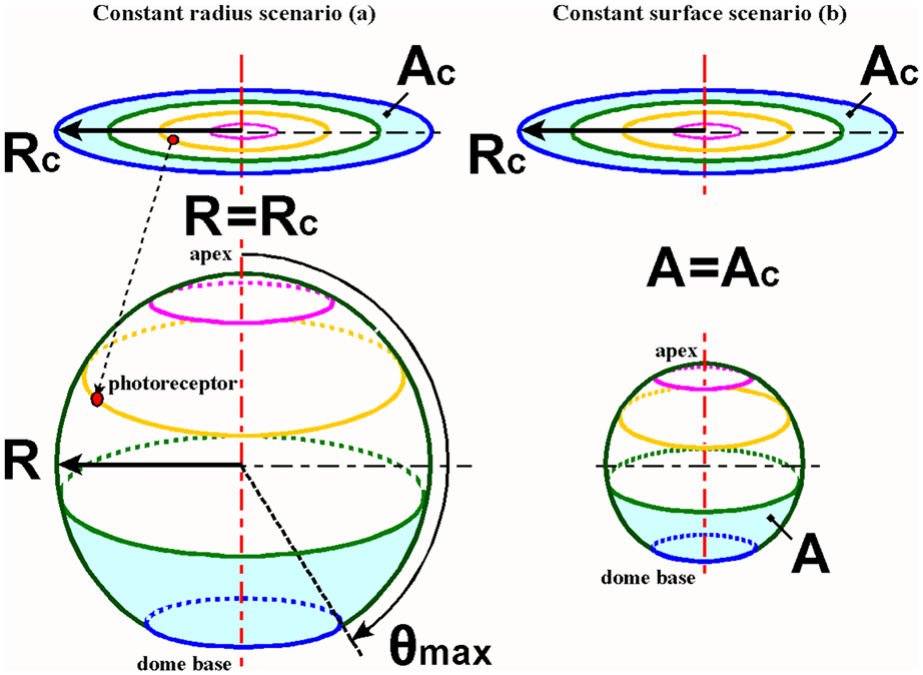
Scale diagram of two evagination scenarios. The initial flat photoreceptor surface with radius *R*_c_ and surface area *A*_c_ is transformed into a spherical photoreceptor organ along two scenarios. The spanned angle *θ*_max_ is 170°. Scenario (**a**) is the constant radius scenario where *R* = *R*_c_ and *A* ≈ 3.9 *A*_c_. Scenario (**b**) is the constant surface area scenario where *A* = *A*_c_ and *R* ≈ ½ *R*_c_. In scenario (**a**), the volume of the sphere is eight times the volume in scenario (**b**)

Equation (1) shows that a smaller sphere radius can provide a greater photoreceptor density and, thus, better spatial resolution. Maintaining the surface of the circular light-sensitive area and the surface of the spherical cap equal during the bulging process results in a constant surface area scenario, with radius *R* becoming increasingly smaller as the spanned angle *θ*max of the spherical cap increases. Both scenarios are illustrated in Fig. 5.

**Fig. 6:**
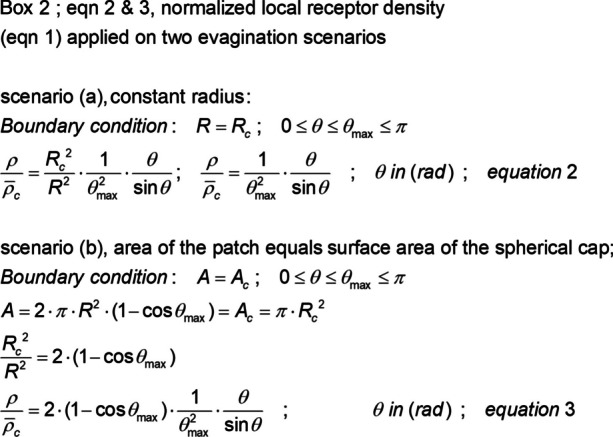
Normalized local photoreceptor density equations. The initial photoreceptor density on the light-sensitive patch is considered to be evenly distributed. Eq. (1) applied to the constant radius scenario *R* = *R*_c_ produces Eq. (2) and to the constant surface area scenario *A* = *A*_c_ Eq. (3)

The corresponding local normalized photoreceptor density equations are shown in Box 2^1^ (Fig. 6).

**Fig. 7:**
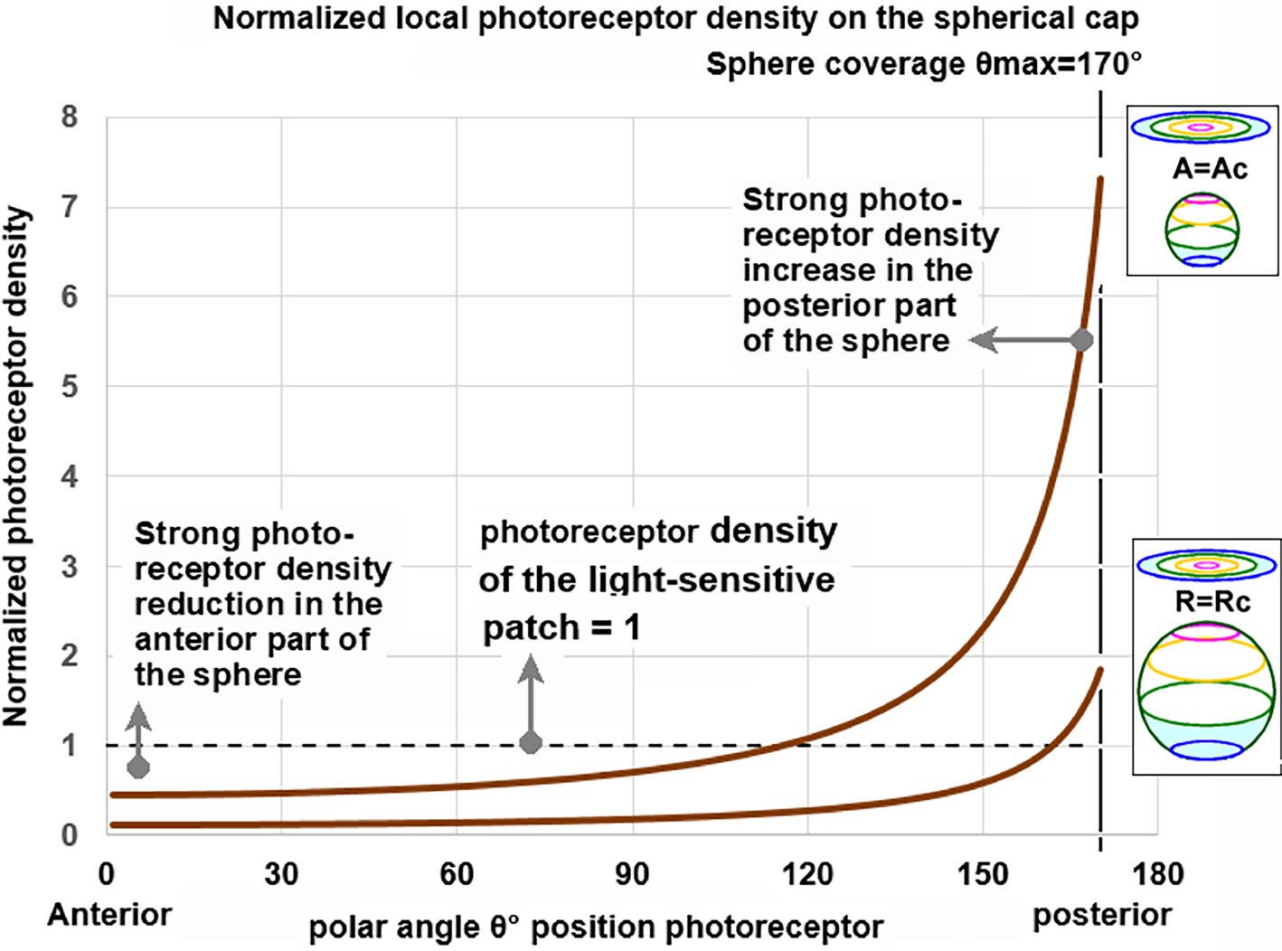
Normalized photoreceptor density profile for two evagination scenarios. A spanned angle *θ*_max_ of 170° is assumed to allow for axons and veins to pass through the retina to the stalk. A normalized local receptor density Eqs. (2) and (3), where angles can be presented in degrees (°) is available in Online Resource 3

The initial photoreceptor density on the light-sensitive patch can be attributed to a normalized value of 1. With increasing evagination, along with an increase in the spanned angle *θ*_max_, a drastic redistribution of the photoreceptors occurs, with a low density in the anterior region and high photoreceptor density in the posterior region of the sphere. Figure 7 uses Eqs. (2) and (3) to report the local normalized photoreceptor density at a spanned angle *θ*_max_ that allows for the passage of axons and veins through the retina to the connecting stalk, which is necessary for the nutrition of the retina and its communication with a coordinating “brain” system.

**Fig. 8:**
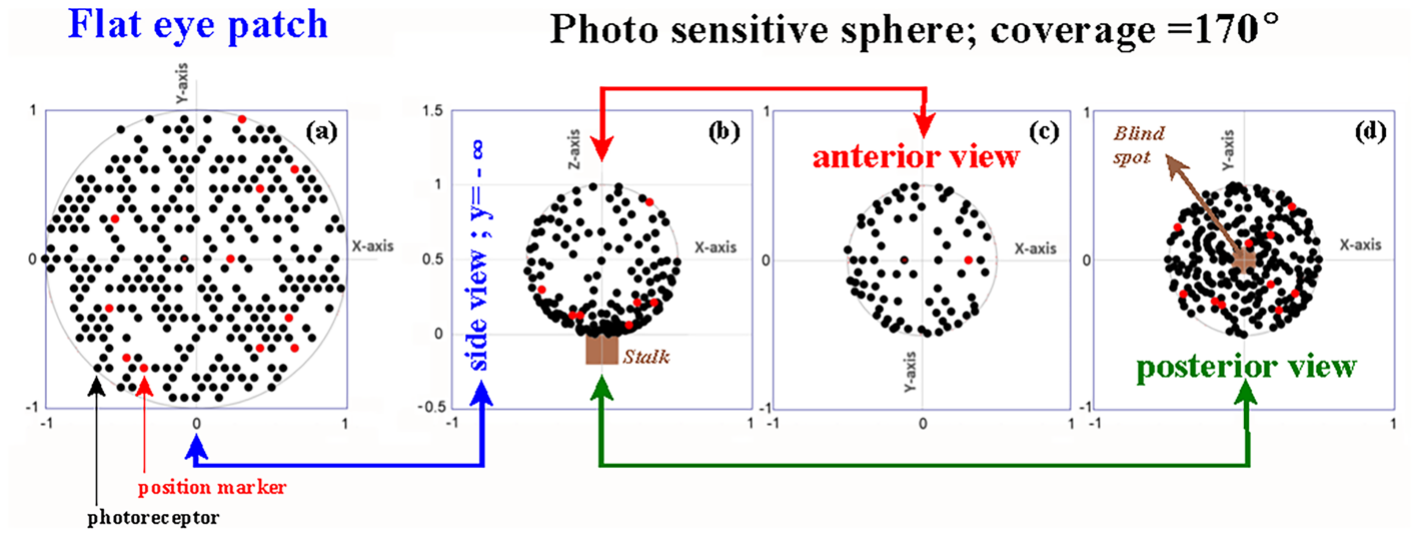
Computer simulation of how the 2D flat photoreceptor configuration distributes spatially across the photo sensitive sphere. The flat light-sensitive patch shown in image (**a**) has 300 photoreceptors randomly placed in a hexagon lattice with 600 positions. Images (**b**), (**c**), and (**d**) give an impression of the individual photoreceptor positions in the constant surface evagination scenario *A* = *A*_c_. The red dots are markers to show how individual photoreceptors from the light-sensitive patch position themselves on the sphere. The spanned angle *θ*_max_ = 170°. Image (**b**) is a side view of photoreceptor positions on the sphere. Image (**c**) shows a plan view of a photoreceptor distribution on the anterior hemisphere and image (**d**) in the posterior part, the posterior hemisphere. In a run of 66 simulations, the anterior photoreceptor distribution equals (*µ* $\approx$ 81; *σ* $\approx 6$) versus the corresponding (*µ* $\approx$ 219; *σ* $\approx 6$) in the posterior part of the retina

In the case of the evagination scenario *R* = *R*_c_, the values range from ~ 0.11 at the anterior part of the sphere until 1.8 at its base, and for *A* = *A*_c_, the values range between ~ 0.45 and 7.3, respectively. In either case, a difference of ~ 16 in photoreceptor density was observed between the top and base of the spherical cap surface. This phenomenon was further illustrated in a computer-simulated impression of the position of the photoreceptors before and after evagination (Fig. 8).

In early evolutionary life, photosensitive organs could only register ambient light levels. Therefore, it does not matter whether the initial photoreceptor cell distribution in the “flat” light-sensitive region is uniform or random. Figure 8 shows the computer simulation of the spatial distribution of the photoreceptor cells from a random 2D flat configuration across the photo sensitive sphere.

To emphasize the important refractive properties of the spherical photosensitivity organ, it is called a lensball from this point on. The equations to calculate photoreceptor position on the circular patch and on the lensball are available in Online Resource 4.

Both analyses (Eq. (1) and simulation) showed that approximately 73% of the photoreceptors were located in the posterior part of the retina.

Because of this gooseneck photoreceptor density profile and the fact that the animal’s body shields the posterior photoreceptor cells from direct light activation, a dominant detection direction arises along the anterior–posterior axis of the lensball for incident light coming from the lateral hemisphere.

Light incident along this axis of the lensball creates a blurred image of the lateral hemisphere, in front of the lensball, on the dense packed photoreceptor cells in the posterior part of the retina. The size of the blur spot depends on the degree of refraction. The photoreceptor density underneath the blur spot determines the spatial resolution of the blurred image. It allows the detection of a rough light–dark environmental pattern in the image. The detectable spatial resolution in this pattern will depend on the regularity, amount, and spacing of the photoreceptors, and the quality of the signal processing neural circuitry. Scanning the lateral hemisphere by movement of the body or lensball lends directional sensitivity to the organ and forms the basis for the first primitive picture of the animal’s surroundings.

## The optical blur spot model

The refraction properties of the lensball and the photoreceptor distribution in the retina determine the maximum spatial resolution at which the outside world can be perceived. As the refractive index of the lensball starts to increase relative to the surrounding medium, it reduces the optical blur spot, and the focal point becomes increasingly closer to the retina, thereby improving the observable spatial resolution. To determine the structure of this blur spot as well as its maximum size, a mathematical model was developed in Box 3 (Fig. 10).

The medium is initially water with a refractive index of 1.33; however, it can also be air with a refractive index of ~ 1.00. The vitreous spherical lensball has an incipient refractive index of 1.34, which means that imaging in water is not yet a factor; however, in air, it already leads to a significant optical blur spot reduction.

In early evolutionary light-sensitive lensball, two effects negatively influenced the unambiguous imaging.

**Fig. 9:**
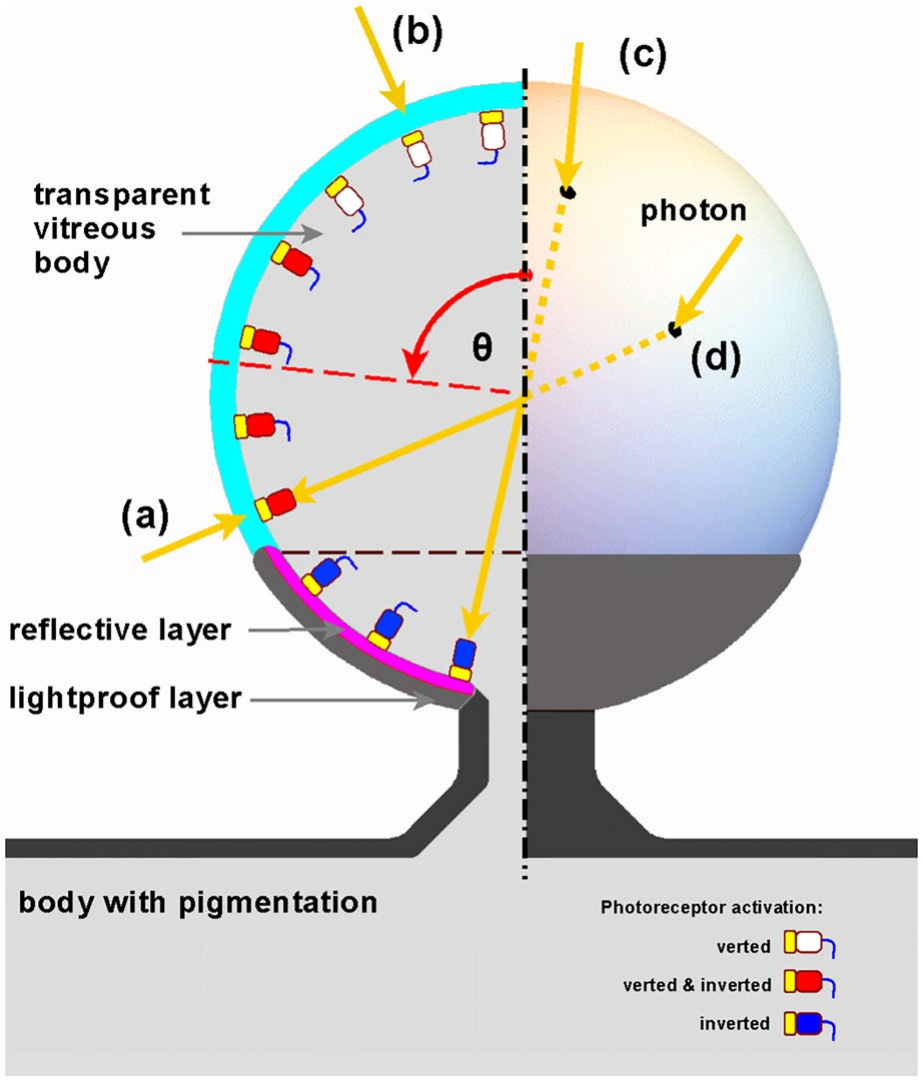
Schematic cross-section of a lensball with a partially developed lightproof outer layer and a transparent vitreous body. The arrows (**a**–**d**) represent how photons can reach a photoreceptor. The photoreceptor is symbolized by a rectangle with a light-sensitive tip. Photons (**a**) and (**b**) directly activate a photoreceptor from outside in a verted manner, whereas photons (**c**) and (**d**) enter the transparent vitreous body and activate a photoreceptor in an inverted manner. Photon (**c**) can only activate the photoreceptor in an inverted manner due to the presence of the lightproof outer layer. An available reflective layer will give photon (**c**), after missing, a second chance to activate. Photon (**a**) and photon (**d**) activate exactly the same photoreceptor. Photoreceptors near the apex cannot be activated in an inverted manner due to shielding by the body and due to refraction if light is coming sideways

The first effect is the activation of photoreceptors in the posterior part of the retina. It can be direct or indirect, producing a photoreceptor signal that is not unambiguously interpreted by the coordinating “brain” system regarding the direction of the light. This problem can be completely resolved by the evolutionary development of a lightproof outer layer on the posterior part of the organ (Fig. 9).

Coverage of the lensball with a lightproof outer layer can occur up to at least halfway up the lensball (polar angle *θ* = 90°) without limiting the anteriorly incident photons from reaching the posterior photoreceptor cells. The lightproof outer layer is now called the “sclera” and the transparent part the “cornea.” The light-sensitive spherical organ with a partially developed sclera and lensball is referred to as the “eye prototype.”

**Fig. 10:**
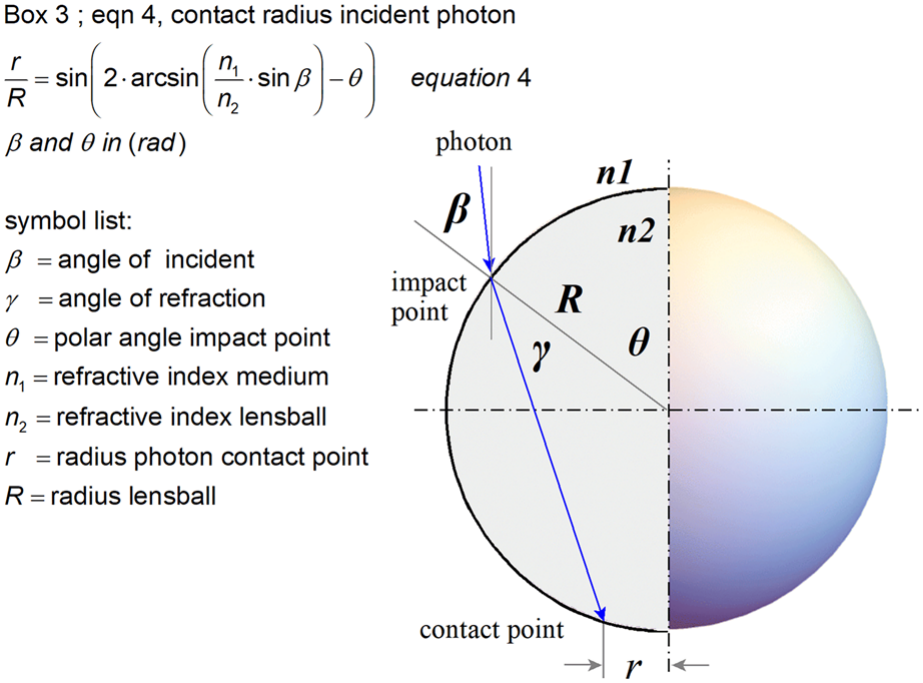
Box 3: scheme for calculating the normalized radius *r*/*R* of a photon contact point on the retina. The lensball is homogenous crystalline and the corresponding refractive index *n*_2_ constant. Snell’s law: *n*_1_ $\times$ sin(*β*) = *n*_2_ × sin(*γ*). For *β* = θ, the photon enters parallel to the polar axis. The polar angle *θ* of the impact point varies between 0 and *π*/2 radians. The photon contact point on the retina has a distance *r* to the optical lensball axis. Derivation of Eq. (4) is presented in Online Resource 5

The second effect is aberration on the posterior retina produced by refraction in the lensball, which causes the margins of the anteriorly incident images to fold back over the central part of the image. This effect becomes more severe as the refractive index ratio *n*_2_/*n*_1_ increases and is therefore analyzed for aspects that may be an evolutionary driving force behind morphological changes, improving the imaging properties of light-sensitive organs. Equation (4) from Box 3 (Fig. 10) is derived to calculate the contact point on the retina for an incident photon as a function of the angle of incidence *β*, polar angle *θ* and refractive indices *n*_1_ and *n*_2**.**_ The position of the impact point is expressed as the radius relative to the optical axis.

In eye lenses from extant fish and in human, the refractive index varies with the position in the lens, low at the lens cortex and high in the lens core, estimates for the central core ~ 1.56 and surface ~ 1.37 refractive indices of the fish eye lens [10]. Human lens estimates for the central core 1.41 and at the lens cortex 1.38. In the eye prototype, immediate compensation for aberration effects is not expected. The modeling assumes a homogeneous refractive index *n*_2_ for the lensball varying between 1.34 and 1.56 [11].

**Fig. 11:**
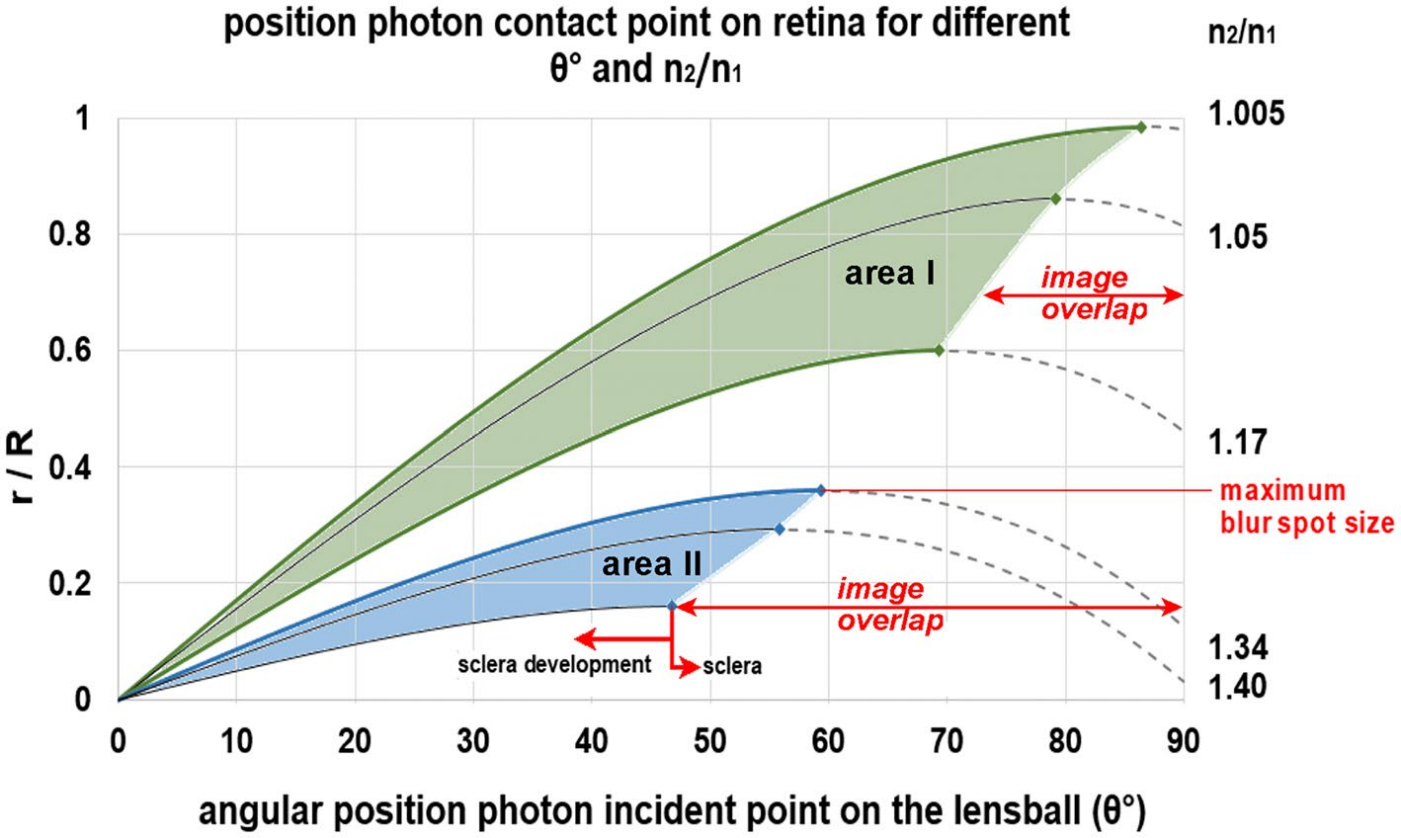
The normalized photon contact radius *r*/*R* as a function of the polar angle *θ* of photon incident point position and the ratio *n*_2_/*n*_1_. The dotted parts of the curves represent the incident angles that cause overlap in the blurred primitive image on the retina. Area I represents the achievable normalized optical blur spot radii of the eye prototype in water. Area II represents the situation in air. The higher the value of *n*_2_/*n*_1_ and the smaller the aperture angle, the smaller the optical blur spot radius and the closer the focus point. The smaller the optical blur spot radius, the better the maximum detectable spatial resolution

For the remainder of this study, *β* = θ, meaning that the incident photon is parallel to the optical axis. The normalized contact point radii are shown in Fig. 11 as a function of the polar angle θ and refractive index ratio *n*_2_/*n*_1_.

With the given *n*_2_/*n*_1_ ratio and increasing angles of incidence θ, the photon impact point moves away from the optical axis until a maximum value is reached, after which it bends back towards the axis, creating a self-overlapping image. Blocking this overlap provided a more readily interpretable image. This may have been the evolutionary trigger to extend the sclera further in the anterior direction to the aperture angle at which the image overlap is eliminated. The rim of the sclera then forms an aperture for the incident light. The aperture angle is defined as the angle between the line from the center of the lensball to the aperture edge and the optical axis. This aperture angle corresponded to the angle shown in Fig. 11, which marked the maximum of the curve.

Depending on the ratio *n*_2_/*n*_1_, this corresponds to a sclera that can extend anteriorly to an aperture angle *θ* between 86° and 71° in water and in air between aperture angles corresponding to *θ* = 59° and 47°.

**Fig. 12:**
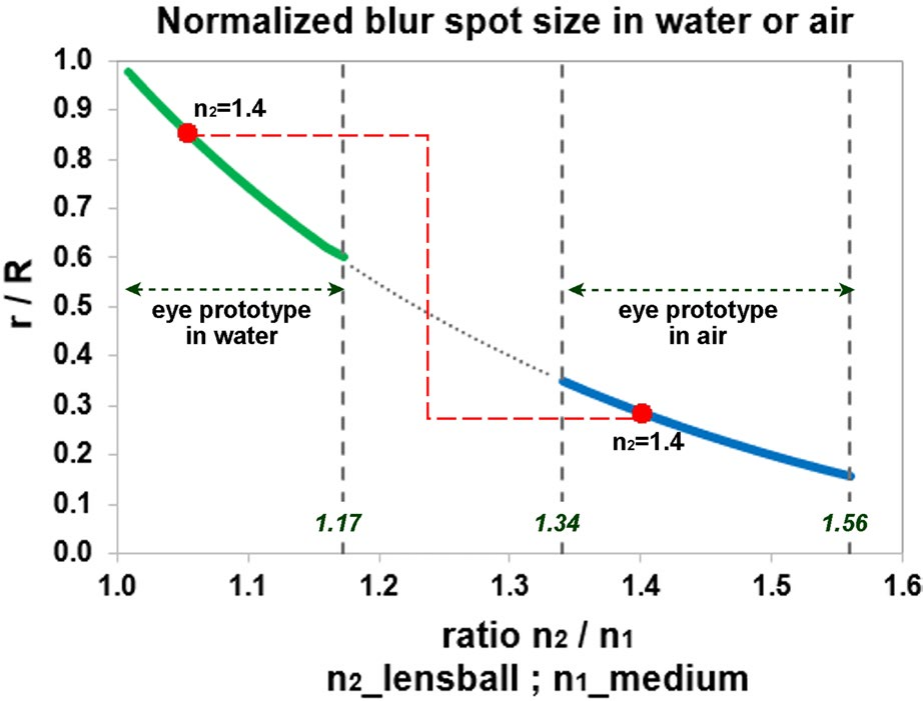
Grafic of the normalized blur spot size as a function of the refractive index ratio *n*_2_/*n*_1_. The descending curve is determined by the maximum optical blur spot radii from Fig. 7. The left solid curve applies to underwater conditions (*n*_1_ = 1.33) and the right solid curve to conditions in air (*n*_1_ = 1) under various values for *n*_2_ according to the crystalline concentration. The dashed line (*n*_2_ = 1.4) marks the transition when the eye prototype crosses the water air boundary

Furthermore, the effect of the refractive index ratio on the normalized blur spot size was analyzed under various biological and environmental conditions (Fig. 12). In water, where the index *n*_1_ = 1.33, the normalized optical blur spot radius (*r*/*R*) varies between 0.98 and 0.59, depending on the crystalline protein concentration, giving a *n*_2_ between 1.34 and 1.56 [11]. Figure 12 shows this process in the normalized maximum optical blur spot curve, where the maximum normalized optical blur spot radii from Fig. 11 are plotted as a function of *n*_2_/*n*_1_.

Sea creatures with a surface-dwelling lifestyle will experience this effect. In air (*n*_1_ = 1), e.g., when the animal is foraging at or above the water surface, the normalized optical blur spot radius can shrink from 0.34 to 0.17 as the crystalline concentration is higher. For a refractive index *n*_2_ = 1.4, the optical blur spot radius decreases by a factor of 2.9 when the eye prototype crosses the surface of the water (indicated by the red dashed line in Fig. 12). The reduction in the blur spot radius averages a factor of 3.2 ± 0.4, suggesting that the fully developed inverted retina may have occurred first in water surface foraging animals. Sea creatures with a surface-dwelling lifestyle will have experienced this effect during evolution and adapt to it with eyes that, swimming at the surface, can see below and above the water at the same time like the extant fish in the Anablepidae family evolved. The eye prototype, with its cup-shaped inverted retina and transparent lensball that functions as a positive lens, allows the animal to perceive its surroundings with a coarse spatial resolution. The smaller the blur spot and the higher the photoreceptor density, the better is the maximum detectable spatial resolution. Reason to develop a calculation model to quantify the quality of spatial resolution detection.

## The spatial resolution model

**Fig. 13:**
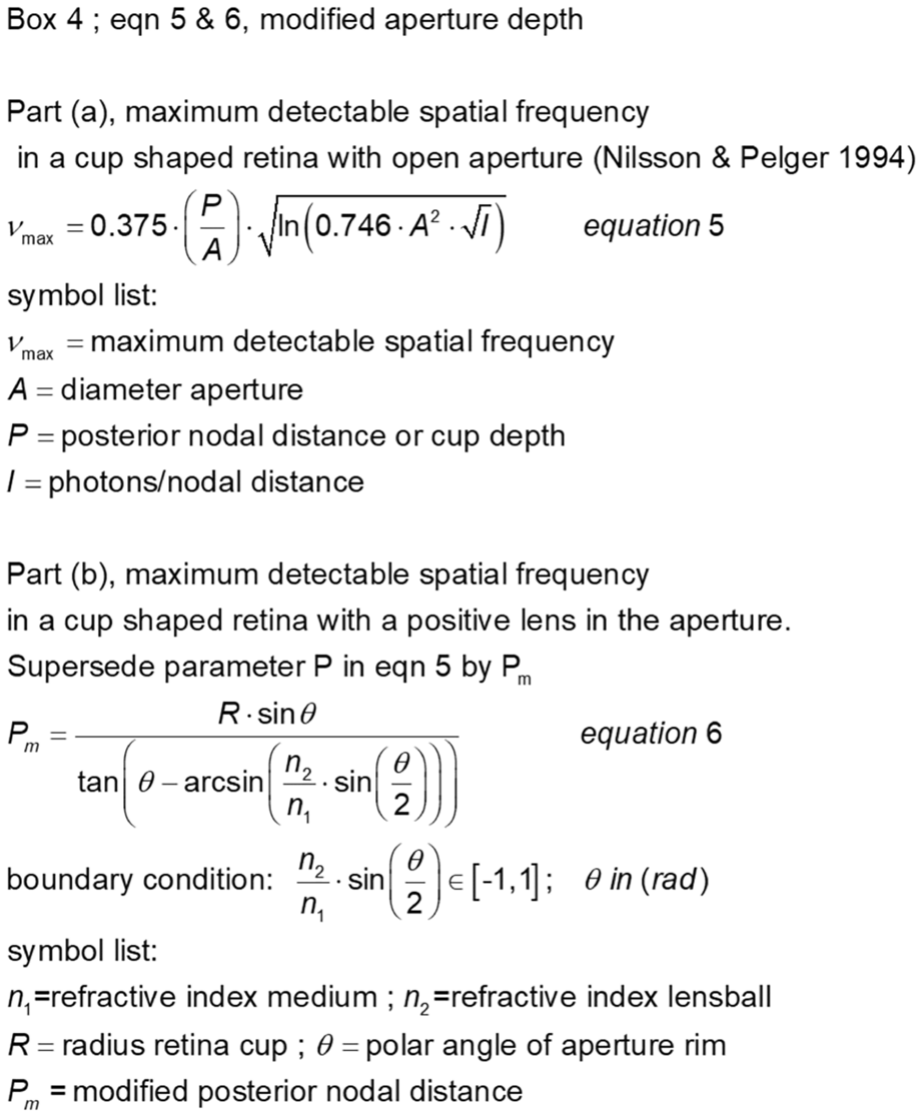
Box 4: maximum detectable spatial frequency for a cup-shaped retina with a positive lens in the aperture. Part (**a**) presents the original equation taken from Nilsson and Pelger [5]. Part (**b**) presents the modified parameter *P*_m_, which accounts for the effect of the positive lens on the maximum detectable spatial frequency and replaces parameter *P* in Eq. (5). Derivation of Eq. (6) is available in Online Resource 6

An improvement in detectable spatial resolution is recognized as an important trigger for eye evolution [5]. Mathematical Eq. (5) (Fig. 13 Box 4) is used to determine the maximum detectable spatial frequency. The equation was designed for an eye prototype with an invaginated cup-shaped retina, and a lens-free aperture for incident light, considering the photon noise. Equation (5) is insensitive to verted or inverted photoreceptor activation. It is assumed that the observable spatial resolution of the retinal photoreceptor mosaic is properly balanced by the optical quality of the rest of the light-sensitive organs.

In Eq. (5), the maximum detectable spatial frequency is directly proportional to the posterior nodal distance (cup depth), which makes it possible to account for the positive lens effect of the lensball in the aperture. By keeping the aperture constant and mathematically modifying the cup radius, the field of view from the posterior pole of the open-cup retina matches the field of view of the lensball retina. The modified posterior nodal distance (modified cup depth) was calculated using Eq. (6) (Fig. 13. Box 4).

**Fig. 14:**
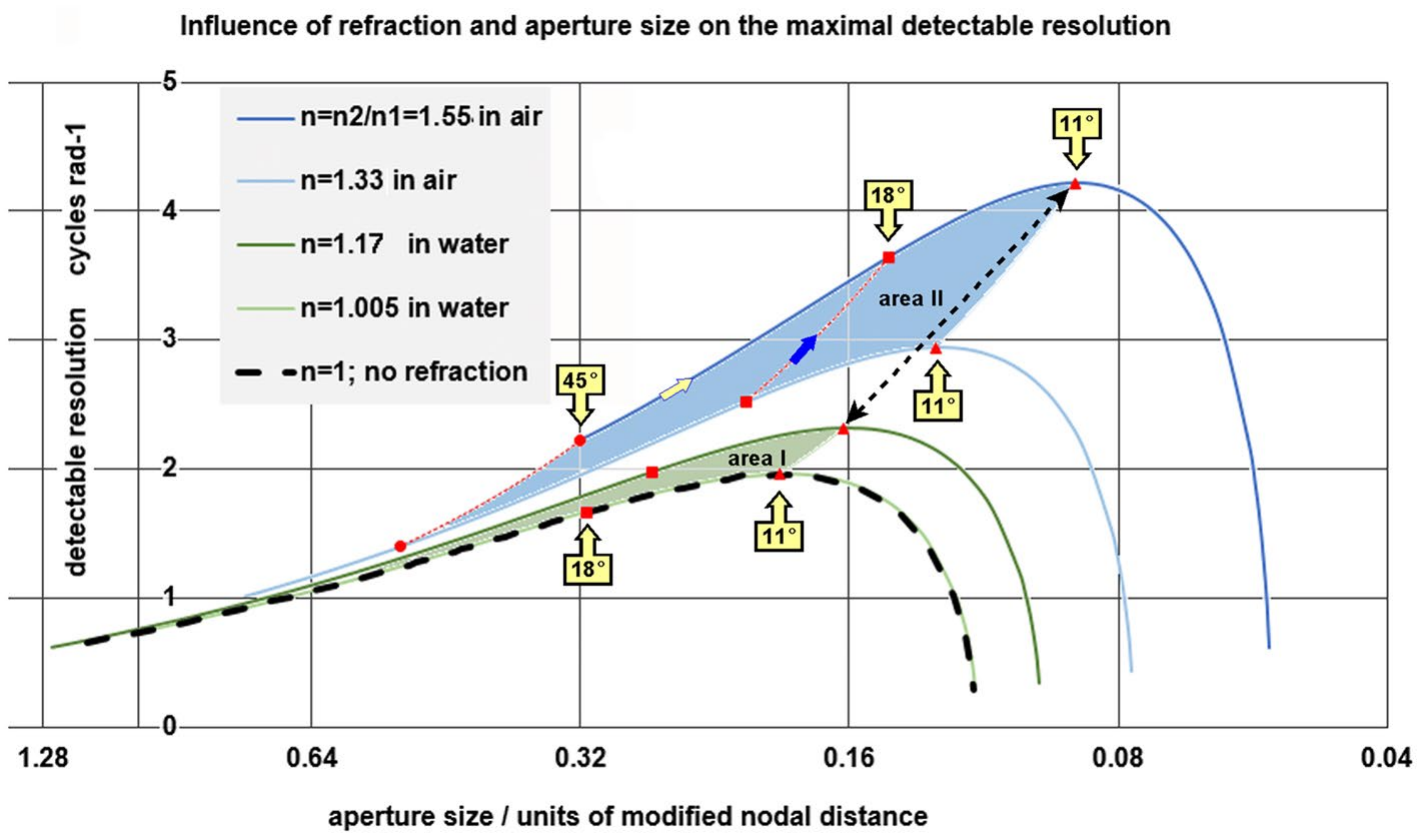
Options for improvement of detectable resolution for the scenario *A* = *A*_c_ and *θ*_max_ = 170°. The influence of the refractive index of the lensball is calculated for a light intensity of 10^4^ photons per normalized surface per second per steradian, taken from Nilsson and Pelger [5] as well as the layout of this figure. The solid lines enclose the area within which the evolutionary optimization of the eye prototype in respect to the spatial resolution can take place. Area I represents the achievable spatial resolution in water. Area II represents the situation in air. The dashed bottom line represents the achievable spatial resolution for the open-cup eye prototype and the lensball eye prototype, provided that both eye types have the same aperture size and the refractive indices ratio *n*_2_/*n*_1_ equals one. The red markers on the lines illustrate progress in detectable resolution as a consequence of aperture angle reduction. The blue arrow points in the direction of an increase in detectable spatial resolution by enhancing the refractive index of the lensball. The bi-directional dashed arrow represents the jump in spatial resolution when the eye prototype emerges above the water

In Fig. 14, the maximum resolution of the lensball is given as a function of the inverse of the normalized cup depth Pm/*A* for a variable refractive ratio *n*_2_/*n*_1_, following Nilsson and Pelger [5]. In water, this provided an increase of approximately 20% in the maximal achievable spatial resolution at an aperture angle of 18°. At an aperture angle of 18°, the spatial resolution in air increases by approximately 46%. The spatial resolution can be further increased by reducing the aperture for incident light, that is, through expansion of the sclera and an increase in the refractive index *n*_2_ until a maximum is reached for an aperture angle of 11°.

A further increase in the maximum detectable spatial frequency is possible by developing extra eye components that increase the amount of light entering the aperture and reaching the retina. This can be achieved by using a positive lens in the aperture or a protruding cornea.

## Eye components

For the sake of convenience, the eye prototype on a stalk is referred to as a “stalk eye” from here onwards. The stalk eye has refractive properties on its spherical surface that allow it to focus. However, in air and with a vitreous body with a refractive index of 1.56, the focal point is still behind the retina, resulting in blurred imaging. For sharp imaging, the focal point must be on the retina, which requires the development of additional components, such as the cornea and/or lens.

To further improve spatial resolution, the eye prototype must evolve a more dense and regular photoreceptor mosaic and a pigmented cell layer behind the photoreceptor layer to prevent blurring by scattering light. This cell layer is a functional innovation of the primitive vertebrate eye [7] and can develop from a single-celled pigmented photoreceptor by cellular differentiation. As described by Gehring [10] into two cell types: a photoreceptor cell specified by Pax6 and a pigment cell specified by the microphthalmia transcription factor (Mitf).

**Fig. 15:**
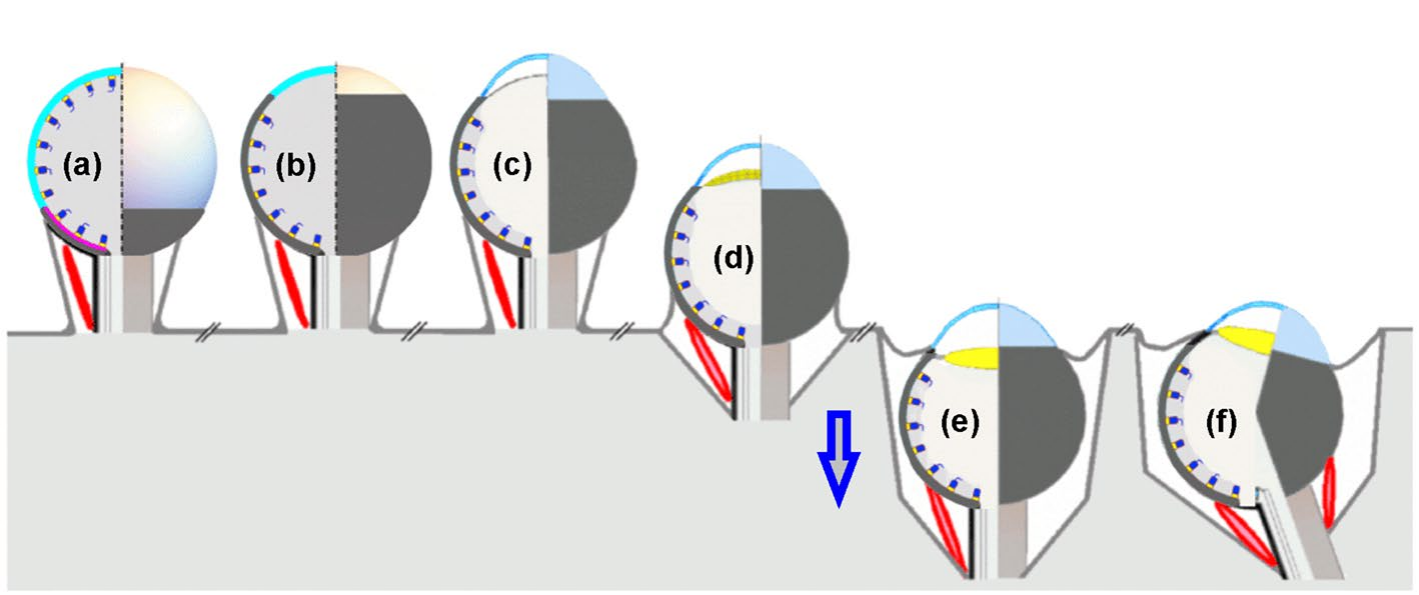
Color-coded hypothetical eye components and their characteristics. (**a**) The stalk containing a channel for axons and veins, with external ocular muscles (shown in red). (**b**) Translucent anterior aperture, free of photoreceptors, coupled to an extended lightproof sclera and a developed RPE layer forming a vertebrate eye prototype. (**c**) Protruding cornea (the eye prototype gets features of a vertebrate eye). (**d**) Proto-lens and retreating apparatus (symbolized by the blue arrow). (**e**) Retreated eye with lens. (**f**) Tilted optical axis positioning the focus point away from the bling spot on the retina

The formation of these components should not conflict with the configuration of the eye prototype and should develop without significant morphological changes. The main eye components are shown in Fig. 15.

- *Extra ocular muscles* (Fig. 15a–f). The eye stalk places the visual organ farther away from the body, and if the stalk is equipped with extraocular muscles, these muscles allow the eye to be oriented. Both features contribute to achieving a wider field of view.
- *Anterior eye aperture as extension of the sclera with tapetum lucidum* (Fig. 15b). The narrow translucent opening bordered by an extended sclera and, eventually a reflective layer behind the photoreceptors in the posterior part of the inverted retina, allows rays of light to penetrate at a right angle through the vitreous body to produce a univocal detailed image on the retina in low-light conditions.
- *Cornea* (Fig. 15c). The few photoreceptors in the transparent anterior part of the retina become inferior to those in the posterior retina, likely prompting their disappearance as the evolution progresses. The transparent epithelial layer covering the anterior retinal layer extends from the sclera and bulges, forming a chamber filled with aqueous humor. This transparent epithelial layer or cornea has a stronger curvature than the lensball and provides an additional refractive surface in air that further reduces the blur spot, contributing to better imaging. However, the cornea does not contribute to improved vision in water. The difference between the refractive indices of water and aqueous humor is too small.

When the cornea and sclera become rigid, mechanical support is provided to the eyeball. Another consequence of corneal stiffness is that the normalized refractive power is fixed. In water, a lens is required to realize a more focused projection on the retina.

In this study, it was assumed that tissue areas with different biological functions were separated by the basement membranes. From an evolutionary perspective, basement membranes are ancient morphological structures already present in sponges and Hydra. During the embryonic phase, they fuse and separate, creating new cell regions with different biological functions [12, 13]. It is therefore justified to use the properties of basement membranes in the evolutionary scenario of the eye prototype and subsequently in the development of eye components that belong to a vertebrate camera eye. The characteristics, basement membrane assembly and basement membrane modification, in tissue sculpting [13] plays a role in the development of the primitive biconvex lens as presented hereafter.

- *Biconvex lens* (Fig. 15d/e). The photoreceptors in the anterior part of the eye prototype disappeared, leaving a transparent epithelial cell layer between the two basement membranes connected to the circulation system. This cell layer can thicken to form a lenticular structure. Consequently, the incoming rays of light converge to focus on the retina, thereby optimizing the observable spatial resolution. This type of primitive lens occurs in the parietal eyes. In the eye prototype, the basement membranes constrict and fuse, which can lead to the separation of the lens from the retina, but they are still anchored to each other by collagen VII fibrils. This collagen VII fibrils could have evolved into suspensory ligaments (Zonule of Zinn). A lens has the advantage of changing its curvature and refractive index, causing it to lose its initial function as a refractive surface for incident light. Maintaining a high concentration of crystalline proteins in the vitreous lensball is no longer required. The separated lens needs its own blood supply only during embryological development what could have led to the separation from the retinal circular system creating the temporary hyaloid canal.
- *Retraction of the eye prototype into the body *(Fig. 15d/e). The protruding soft-tissue stalk eye in the predecessor of vertebrates is vulnerable and, therefore, probably retracted into the body, while the eyeball tilt, rotation, and field of vision are retained.

Note that a structure that generates an additional shrinkage of the aperture for incident light, such as an iris, would be beneficial for the visual resolving power, but would not be necessary for the functioning of the eye prototype.

## Results

### The photoreceptor density model

The developed local photoreceptor density equation predicts low-density anterior and high-density posterior, allowing the posterior retina to develop into an inverted retina sensitive to incident anterior light, creating the basis for detectable spatial resolution.

### The optical blur spot model

The model offers two triggers for scleral development and a trigger for aperture clearance of light-sensitive cells.

Prevention of photoreceptor signals that the coordinating “brain” system cannot unambiguously interpreted (Fig. 9) by coverage of the posterior part of the lensball with a sclera from stalk (θ ~ 170°) to an aperture with a polar angle *θ* ≤ 90°. Annihilating the negative effects of aberration can be achieved by extending the sclera further in the apex direction up to a polar angle that corresponds to the maximum blur spot radius, depending on the environmental air (59° ≤ *θ* ≤ 47°) or water (86° ≤ *θ* ≤ 71°).

### The spatial resolution model

The spatial frequency model of Nilsson and Pelger [5] for an open-cup retina can be used for a lensball retina with a positive lens effect in the aperture by modifying the pit-depth parameter. The equation for calculating the modified pit-depth is shown in Box 4. The spatial frequency model allows for further extension of the sclera in the apex direction up to a polar angle of *θ* = 11°, which corresponds to the maximum detectable spatial frequencies in Fig. 14. This angle is independent of the air or water environment and refractive index of the lensball.

### The eye prototype

**Fig. 16:**
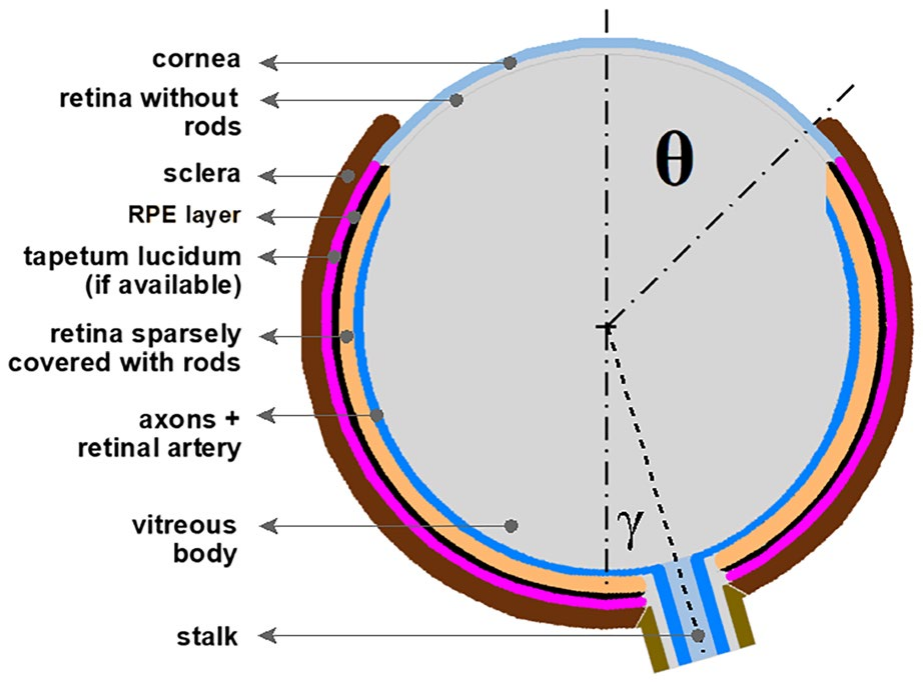
Color-coded schematic overview of the eye prototype. The cornea and the transparent retina without rods closely cover the vitreous lensball; the retina is sparely covered with rods and has a RPE cell layer to absorb stray light. The tapetum lucidum optional in low-light conditions. Polar angle *θ* determines the aperture. The angle *γ* between optical axis and the optic disc center. The blue lines the axons towards the coordinating “brain” system

The eye prototype was generated from a scenario driven by continuously logically improving the detectable spatial resolution with small incremental steps in the morphological change process (Fig. 16).

The eye prototype without lens and cornea but with a fully developed sclera, corresponding aperture, potential tapetum lucidum, RPE cell layer, and an aperture with a polar angle of *θ* = 11°, has a detectable spatial resolution in air between three and four cycles/rad and in water around two cycles/rad, proving that the evagination scenario for eye development could explain the origin of the inverted retina.

**Fig. 17:**
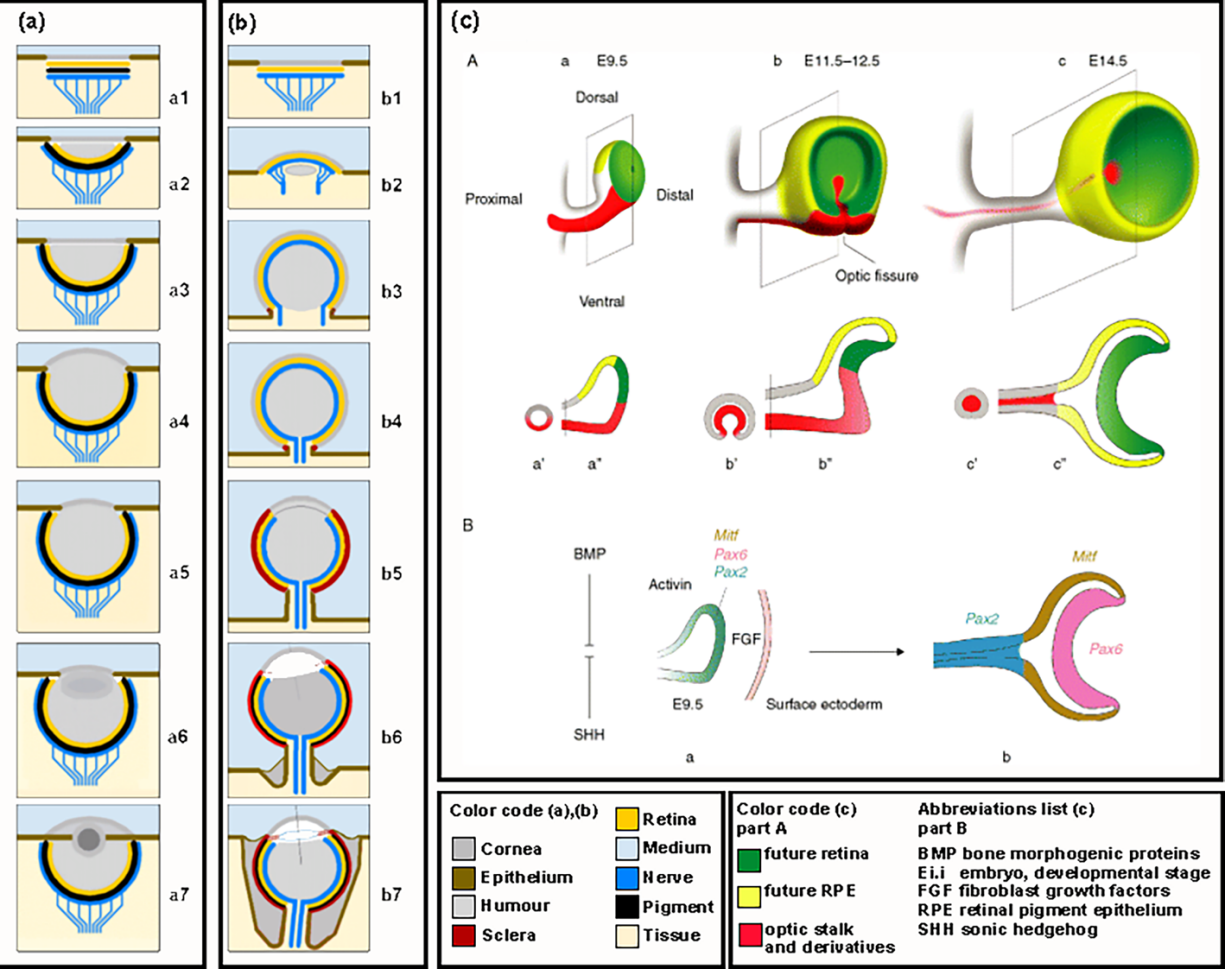
Schematic comparison of the hypothetical evolutionary pathways of two different eye prototypes. (**a**) Steps 1–7 of the developmental pathway of the cephalopod proto-lens-eye, redrawn from Nilsson and Pelger [5]. (**b**) Developmental steps of the vertebrate predecessor eye prototype. b1 The initial light-sensitive patch has a retina with primitive photoreceptors and not yet a screening pigment layer compared to (a1) in the evolutionary sequence given by Nilsson and Pelger [5] and Land and Nilsson [1]. b2 Different evolutionary pathways for evaginating photoreceptor patch compared to the invaginating patch (a2). b3 Development of posterior retina. b4 Posterior inverted retinal activation, start detectable spatial resolution and imaging. b5 Fully developed inverted retina, lightproof sclera and clear aperture shaping the vertebrate eye prototype. b6 Speculative, primitive lens and screening pigment layer development. The optical axis tilts with respect to the stalk axis. Starting retraction of the eye prototype into the body. b7 Speculative, fully developed eye prototype in the predecessor of the vertebrates. **c** Visualization the areas where the tool kit genes Pax2 and Pax6 come into action in the human embryo. It illustrates the close involvement of the Pax2 gene in optical nerve tube development and the resulting optic disc in the retina (red area) and the involvement of Pax6 gene (green area) in the retina cup development in the vertebrate eye. From Bharti [14], used with permission from John Wiley and Sons

Figure 17 presents the hypothetical developmental pathway for the inverted retina and lens-eye components of the vertebrate eye prototype, parallel to the accepted developmental pathway of the cephalopod proto-lens-eye. It shows the embryological formation of the vertebrate eye and the involvement of toolkit genes Pax6 and Pax2 [14].

## Discussion

It is likely that an inverted retina evolved in vertebrate predecessors from a primitive spherical transparent light-sensitive organ on the stalk. Light-sensitive organs on the stalk are a common feature in nature. The spherical light-sensitive organ has a retina that is activated by direct and inverted incident light. This study shows that small configurational modifications triggered by spatial resolution optimization give rise to an inverted retina in a functional eye prototype by using incident light for all vision functions rather than direct light. Incident light captured by the concave posterior part of the retina allows for imaging, whereby the retina in the posterior part is superior to that in the anterior part. Configuration (b5) in Fig. 17 shows the eye prototype.

The optimum aperture was independent of the ratio *n*_2_/*n*_1_ and had a polar angle of 11°. This suggests that, for the stalk eye, scleral development has priority over other evolutionary options for spatial resolution improvement. The maximum resolvable spatial frequency that can be achieved with the optimum aperture varies in water between 2 and 2.3 (cycles per radian) and in air between 3 and 4.3 (cycles per radian), depending on the protein concentration and, therefore, the refractive index *n*_2_ of the vitreous body. A deep-sea isopod has a maximum resolvable spatial frequency of 1.9, and it is 3.6 for Nautilus [1].

Another reason is that, in the spherical eye prototype, the photoreceptor density across the sphere increases by a factor of ~ 16 from anterior to posterior. This factor is independent of the chosen evagination scenario but is based on the assumption that photoreceptor cells retain their position in the support matrix.

The assumption that the photoreceptor cells retain their position in the support matrix upon bulging of the light-sensitive organ, and therefore become unevenly distributed, is vulnerable because there may be an early evolutionary genetic mechanism that keeps the photoreceptor cells evenly distributed across the surface area at all times during the shape change process. This mechanism is not expected to occur in an organ sensitive only to light intensity and direction, in which detectable spatial resolution does not yet play a role. However, it is to be expected in an eye prototype that already has imaging capabilities, as a more uniform retinal photoreceptor pattern directly affects the image resolution positively.

Photoreceptors located in the convex anterior part cannot contribute to detectable spatial resolution because the spatial angle of view is close to 180°; therefore, the detectable spatial resolution is zero. The processing of signals coming from the anterior part of the retina loses its function, and the corresponding light-sensitive cells disappear evolutionarily by random genetic drift, producing a heritable phenotypic variation. According to Masel [15], this typically requires a few generations of isolated populations. Also, selective pressure to reduce the high energetic costs maintaining anterior photoreceptors and neurons functioning may be a reason for disappearing [16].

We can expect that tidal zones and coastal areas are the primary areas for small isolated populations. The remains of water-bound plants and animals are washed up on the coast and banks of rivers and waterholes. These nearshore zones are expected to be already highly bioactive during the Precambrian period.

An increase in photoreceptor density in the posterior part of the retina and a more regular distribution of photoreceptors will further increase spatial resolution. In this regard, a hexagonal configuration is the most obvious because it yields the densest photoreceptor bunching and thus the smallest spacing between photoreceptors, as observed in contemporary animal-eye retinas.

The soft-tissue stalk eye is vulnerable to physical damage and, therefore, is probably retracted into the soft-tissue animal body while still being able to scan the animal’s surroundings. During the Cambrian, hard internal and external body parts developed in various animal species and embedded the vertebrate eye into the skull. Eyes and optic stalks were better protected.

There are no known fossil finds from the Precambrian or Cambrian that support the stalk eye hypothesis in vertebrates. Accepting that stalk eyes could be a precursor to the vertebrate eye will contribute to the interpretation of eye characteristics in Precambrian fossils. Evidence of soft-tissue eye prototype on a stalk can possibly be found in the chemical traces of retinal molecules beyond the body imprint.

The amphioxus eye, with its inverted interpreted retina, does not fit the stalk eye hypothesis, unlike the more advanced bulging eyes of conodonts [17]. The eye of the hagfish has morphological phase 3 characteristics (Fig. 2) because it comprises an inverted retina around a vitreous spherical body. The difference with phase 3 is that it has a retinal pigment epithelium that starkly lacks pigment [18]. It is likely that the eye was retracted into the body without maintaining the optic stalk. The eyes in the larval phase of lamprey are similar to the eyes of hagfish, but migrate outward with the development of the lens and cornea.

The stalk is a new structure that requires a genetic basis that may be provided by the Pax2 gene, which plays an important and tightly delineated role in embryonic development of the optic tract in the vertebrate eye [19]. In his review of the various genes involved in eye development, Fernald [20] indicated that Pax2 is expressed within the embryonic vertebrate eye only, and not in other phyla. This is in contrast to Pax6, which is active in all phyla. Fernald [20] postulated that Pax6 is almost certainly active in the precursors of vertebrates, shellfish, insects, and cephalopods; however, it is unclear whether the same is true for Pax2. The Pax2 gene belongs to the early evolutionary genetic toolbox along with the Pax6 gene, which makes it likely that Pax2 is involved in the development of the eye prototype on a stalk. No direct conflict between embryonic development and morphological outcomes was observed. The light-sensitive patch is of ectodermal origin and, therefore, the retina in the eye prototype. The ectodermal retina was probably later transformed into neuroectodermal tissue.

There are vertebrate fish, like Idiacanthus, that develop full-stalk eyes during their larval phase to increase food searching capabilities [8]. These types of larval eyes can potentially elucidate the genetic process and explain the genes responsible for the larval eye stalk. If the involvement of the Pax2 gene is demonstrated, this would serve as a strong indication that this developmental pathway may also have occurred during an early evolutionary phase and genetically conserved. Otherwise, these fish stalk eyes represent a more recent evolutionary development.

The future retinal area on the optic vesicle invaginates as an incomplete spherical cup with a wedge-shaped opening, which then deforms into a spherical cup during retinal fissure closure.

Thus, retinal development in the vertebrate eye is the result of two different topological actions: distal indentation and ventral indentation.

Distal indentation alone does not lead to the development of the optic disc as a passageway for axons in the retina. A functioning eye prototype is impossible.

Pax2 directs the ventral notch of the nascent optic vesicle and results in development of the optic stalk and optic disc in the vertebrate retina (Fig. 17c. [14]).

Topologically, exclusively ventral notching of the optic bud can lead to the development of a functioning eye prototype, which demonstrates the important topological role of Pax2.

An intriguing question is whether the present mammalian eye with its optic stalk and extraocular muscles is a further developed eye prototype on a stalk that, due to its fragility, was gradually retracted into the animal’s head while retaining its pointing ability. In this case, the contemporary optic stalk in the mammalian eye could be a functional successor of the eye prototype stalk.

## Conclusion

This study shows that verted and inverted retinas probably developed along two different morphological processes. Each process as the starting point for a different eye prototype development scenario. An invagination process results in a verted-light-facing retina in cephalopod eyes and an evagination process, resulting in an inverted retina in eyes of vertebrates.

*Proposed evolutionary scenario for the inverted retina*: an evaginating flat light-sensitive area with spaced-out photoreceptor cells in a light-facing orientation developed into a protruding spherical transparent light-sensitive organ covered with light-facing photoreceptors with elongated axons running on the inside, resulting in a photoreceptor density that is lowest in the anterior cap of the sphere and increases to the highest in the posterior part. Photoreceptor activation by direct light is blocked by lighttight scleral development on the outside of the sphere, resulting in a concave retina capable of increasing spatial resolution detection without the need for morphological retinal modifications and making the sparse photoreceptors in the anterior cap become obsolete and disappear.

The proposed scenario, arising under evolutionary pressure to achieve the highest possible detectable spatial resolution, has the full potential to explain the origin of the inverted retina.

This study suggests that a primitive inverted retina in the predecessor of vertebrates is of ectodermal origin and available before neurulation occurred.

## Supplementary Information

Below is the link to the electronic supplementary material.

Supplementary file1: The role of body pigments, retinal pigment epithelium (RPE) cells, refraction and the conditions for spatial resolution detection in the evolutionary evagination scenario of the inverted retina and the principles for the presented modeling (PDF 695 KB)

Supplementary file2:  Normalized local photoreceptor density (Eq. 1) where angles can be entered in degree. The presentation of Eq. 1 in degrees is given (PDF 97.9 KB)

Supplementary file3:  Normalized photoreceptor density Eqs. (2 and 3) where angles can be entered in degree (PDF 183 KB)

Supplementary file4:  Equations to transform photoreceptor position on the circular patch into the position on the lensball (PDF 270 KB)

Supplementary file5:  Eq. 4 is used to examine the image structure on the retina and to calculate the maximum blur spot size (PDF 257 KB)

Supplementary file6:  Derivation of the modified cup depth to analyse the influence of a positive lens in the aperture of a retina cup on the maximum detectable spatial resolution (PDF 214 KB)

## Data Availability

No datasets were generated or analysed during the current study.

## Footnotes

1 Due to the mirror symmetry of the evagination and invagination process, Eq. (1) can be applied to both sphere geometry and cup geometry.
