# Supplementary material for: A possible origin of the inverted vertebrate retina revealed by physical modeling
Source: J Biol Phys. 2024 Aug 3;50(3-4):327–49. doi: 10.1007/s10867-024-09662-6 (PMC11490472; doi:10.1007/s10867-024-09662-6)
Supplement: Supplementary file 1 — Supplementary file1: The role of body pigments, retinal pigment epithelium (RPE) cells, refraction and the conditions for spatial resolution detection in the evolutionary evagination scenario of the inverted retina and the principles for the presented modeling (PDF 695 KB) [file 10867_2024_9662_MOESM1_ESM.pdf]

A possible origin of the inverted vertebrate retina revealed by physical modeling; Journal of Biological Physics; Jan M.M. Oomens; Independent researcher; oomens-science@ziggo.nl

The role of body pigments, retinal pigment epithelium (RPE) cells, refraction and the conditions for spatial resolution detection in the evolutionary evagination scenario of the inverted retina and the principles for the presented modeling.

Principles

The principles in this study on pigmentation and light directivity can be summarized as follows:

1. The RPE cell will not be used in the evolutionary scenario of the inverted retina in the ancestor of vertebrates.
2. RPE cells are introduced into the evolutionary scenario as soon as a logical need for them arises.
3. Light directionality detection can be achieved by blocking light, shielding by body pigmentation and by refraction.
4. Refraction of light and body pigmentation are enough to achieve light direction detection in the bulging light-sensitive patch.
5. Spatial resolution detection is not possible on a convex photoreceptor surface.
6. Spatial resolution detection is possible in a convex curved surface with photoreceptors sunken in funnel-shaped ommatidia.
7. Spatial resolution detection in a one chamber eye needs a concave retina.

The principles are discussed in separate paragraphs.

Contents

1. The role of pigments and RPE cells in light direction detection

2. The role of refraction in light direction detection

3. The conditions for spatial resolution detection

1. The role of pigments and RPE cells in light direction detection

In retina studies of living animals, the photoreceptor is always accompanied by an RPE cell. The RPE has several functions namely, light absorption, epithelial transport, spatial ion buffering, visual cycle, phagocytosis, secretion and immune modulation. The question is whether this package of functions was already necessary in the pre-cambium period. Probably not for the wide spread and simple ciliary photoreceptor cells (rods) in the light sensitive patch. Rods are directly connected to the signal processing centre by an axon and can respond to a single photon. Colour detection and neural filters are regarded as a later development of the simple retina and expelled from this study.

Fig. I Phases of the evagination scenario for inverted retina development

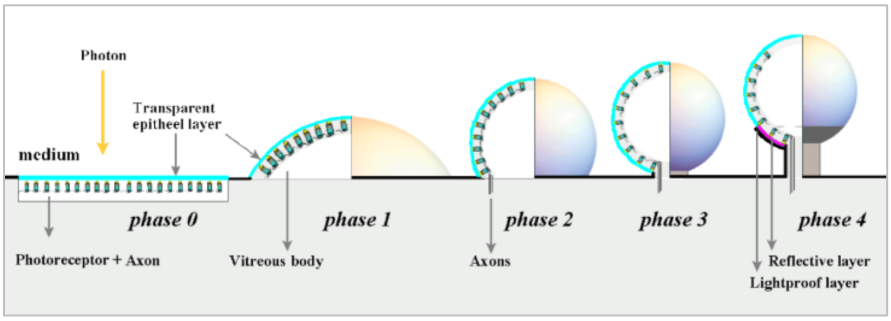

It is assumed that the vertebrate ancestor had pigmentation in the body as shielding for destructive influence of ultra violet radiation and will absorb

all light. The need for light shielding others than by body pigments arises in phase 4 but does not need immediate development of RPE cells. Refraction effects and light blocking by the lightproof layer are sufficient. Fig. II shows how the patch in phase 0 is build up.

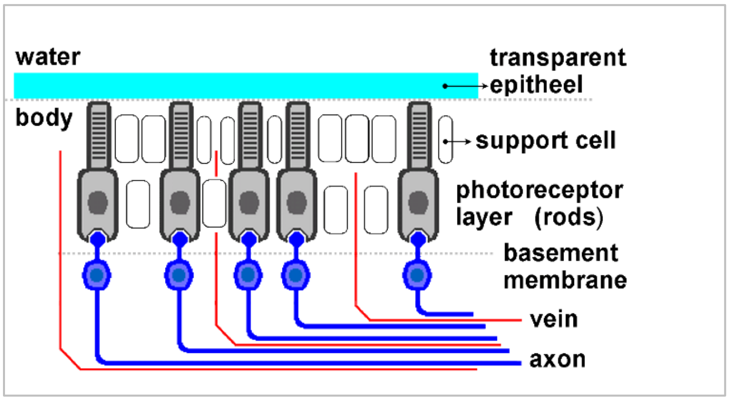

Fig. II Cross section of the light sensitive cell layer (light-sensitive patch). The space between photoreceptors is filled up with transparent support cells. The photoreceptor layer is sandwiched between basement membranes. Light enters from the water side and doesn't have to cross the veins and axons layer. Light that is not activating a photoreceptor will be absorbed by the pigmented body

The prerequisite for this study is therefore that body pigmentation is available. RPE cells are introduced into the evolutionary scenario as soon as a logical need for them arises. Three studies were used to substantiate these principles

Ramón Martínez-Morales [5] hypotheses a tentative model for the evolutionary origin of pigmented cells. An ancestral ectodermal cell containing two types of pigments gave rise to invertebrate rhabdomeric photoreceptors. In the chordate phyla, the same ancestral ectodermal cell gave rise to two independent cells, cell with visual pigment and a cell with dark pigment. See Fig. III.

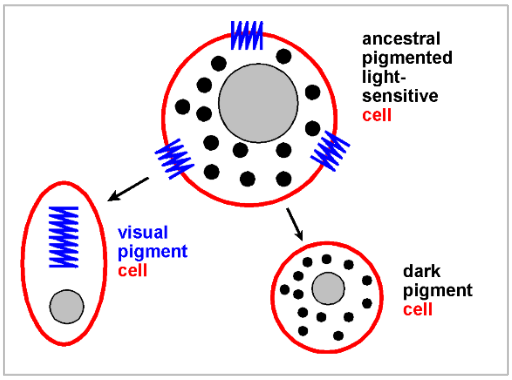

Fig. III Model for the evolutionary origin of pigmented cells (redrawn from [5]). An ancestral pigmented cell has visual pigment on the outside to detect ambient light level and dark pigment to absorb light to protect the cell nucleus for destructive ultra violet light

Albalat [1] concludes that the retinoid cycle machinery was not present in the ancestor of vertebrates and therefore a functional innovation of the primitive vertebrate eye. Only ciliary photoreceptor cells and ciliary (c)-opsins were already present in the photoreceptor system of the ancestor of the vertebrates.

Sustained vision requires continuous regeneration of opsins via the retinoid cycle, also called the visual cycle. The retinoid cycle in the RPE cell provides a continuous supply of visual chromophore independent of light. In addition to the classical retinoid cycle, retinal G protein-coupled receptor (RGR) in the RPE and Müller cells contributes to the production of visual chromophore, particularly in response to the level of light exposure [1]. That raises the question how the visual cycle for the photoreceptor rod in the primitive light sensitive layer worked. The paper of Kusakabe et al [3] point out that the entire process of the visual cycle can occur inside the photoreceptor cells and that the visual cycle components are also present in surrounding non-photoreceptor cells. It is supposed that the surrounding non-photoreceptor cells can also produce dark pigment.

Chosen principles role of pigments and RPE cells in light direction detection:

1. The RPE cell will not be used in the evolutionary scenario of the inverted retina in the ancestor of vertebrates.
2. RPE cells are introduced into the evolutionary scenario as soon as a logical need for them arises.

2. The role of refraction in light direction detection

Refraction in a transparent lensball has the same effect on shadow formation as blocking light by an opaque object.

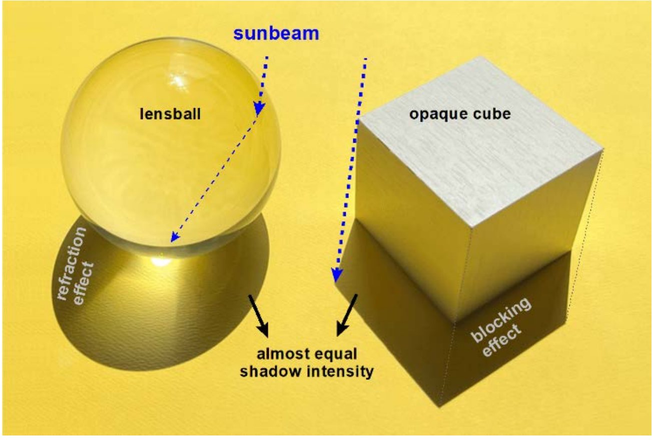

Fig. IV Photo, made by the author, of the shadow effect of a transparent lensball and of an opaque cube in sunlight. The lensball is homogeneous with a refraction index that is constant throughout the lensball. The path of a sun beam is shown by dashed parallel arrows.
The sun beam on the lensball is refracted and producing a space inside and underneath the lensball free of sun light. I call this a light free space with the available shadow area as the only visible boundary

Imaging that a person gets protection against the sun in the light free space underneath a big enough transparent lensball and careful avoiding the spot with high concentrated sunlight.

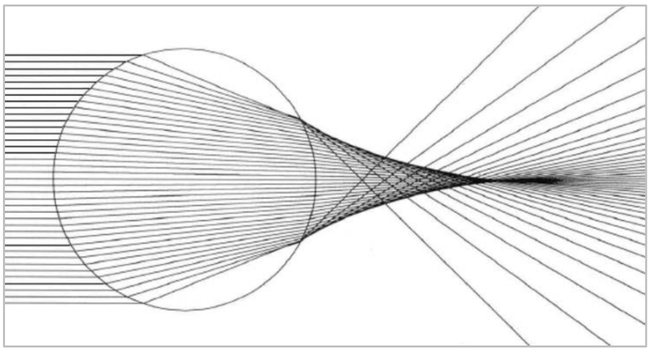

Fig. V Schematic diagram of the refraction pattern by a fan of parallel laser beams on a transparent lensball. Redrawn from [2]

A transparent lensball is illuminated with a fan of parallel laser beams. The refraction pattern is shown. The region where light leaves the sphere is of importance. This area forms in a concave retina the blur spot.

Insight from Fig. IV-V can be applied to the dome shaped retina in phase 3 of Fig. I.

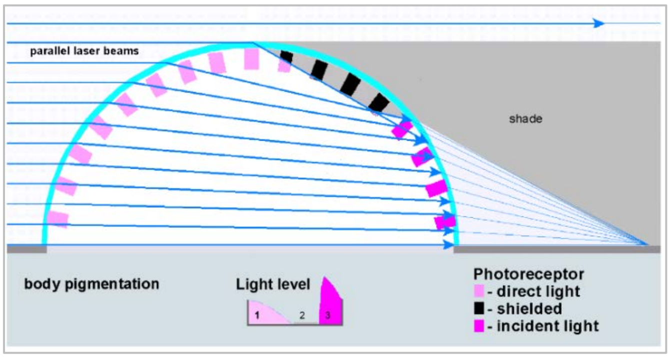

Fig. VI Scheme showing a cross section of a transparent dome, covered with a retina in which the photoreceptors face outwards, illuminated by a fan of horizontal and parallel laser beams, showing how a shaded space results from the effect of light refraction in the dome. The light free space is not visible. In the retina, areas are created with different light levels as shown in the inset

The beam can activate a photoreceptor directly (verted manner) or indirectly on the opposite side of the dome (inverted manner).
The signal processing brain can distinguish between three type of receptor signals, see fig. VI:

- receptors receiving direct light level (light magenta)
- receptors shielded and not activated (black)
- receptors receiving high intensity incident light (Intense magenta).

In development phase 1 and 2 of Fig. I, that difference should be enough for an organism’s nervous system, capable of comparing information from various photoreceptor cells, to determine the direction of the light. This is also shown in branch (b) of Fig. VII (figure 1 in the paper). Photons not absorbed are leaving the dome.

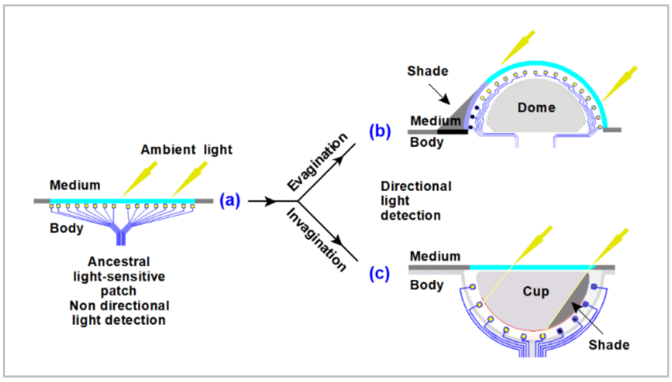

Fig. VII Dome and cup configuration create directional light detection

Nilssons [6] hypothesis that "Directional light sensitivity may have started as a by-product of protective body pigmentation" is symbolised in Fig. VII, part (c) by the shade in a retina cup coming from blocking light.

In Fig. VII, part (b) the shade from a transparent retina dome results from light refraction effects as shown in Fig. IV.

In both cases some photoreceptors are not activated allowing the brain to interpret light direction. When light is coming perpendicular to the cup or dome in Fig. VII, it will activate all photoreceptors and not on the opposite body side.

Imagine a full developed light-sensitive organ, as presented in phase 3 of Fig. I, in turbid water where there is an equal level of light around the organ. The light coming from all sides to the sphere is shown in Fig. VIII. In that case body shielding results also in retina areas with different illumination.

Photoreceptors in the deeper concave posterior part of the retina are shielded for direct light by body pigment and illuminated by incident light, coming from anterior direction, making detection of spatial resolution possible. This is symbolised by the green-yellow-green inset in Fig. VII. As a consequence of the body shielding, the photoreceptors in the top part of the anterior retina are shielded for incident light. A brain capable to distinguish between the three different illuminated retina areas can use the retina area with only incident light activation to perceive a part of the background with a certain spatial resolution.

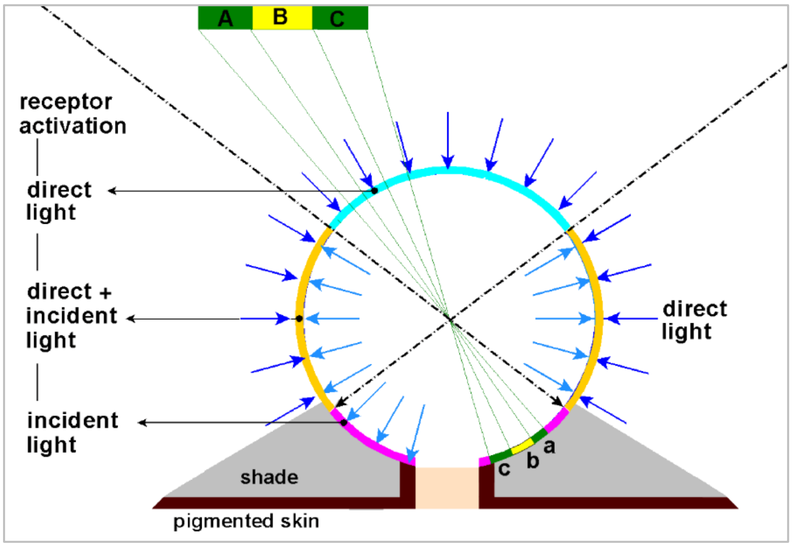

Fig. VIII Effect of body pigmentation on light direction detection and detectable spatial resolution in phase 3. Three type of retina illumination areas can be distinguished:

- a direct light area
- a direct + incident light area
- an incident light area

The black dashed arrows mark these areas

The background part marked ABC is projected blurred on the concave posterior retina.
The organ can detect light intensity, light direction and a blurred background pattern. The level of the spatial resolution depends mainly on the refractive indexes of lensball and medium.

Chosen principles for light directionality detection:

3. Light directionality detection can be achieved by blocking light, shielding by body pigmentation and by refraction.
4. Refraction of light and body pigmentation are enough to achieve light direction detection in the bulging light-sensitive patch.

3. The conditions for spatial resolution detection

Define spatial resolution as a measure of the smallest object that can be resolved by the sensory organ.

How does that work for a single photoreceptor in a convex curved retina. Figure VI left part provide an explanation for the conclusion that a convex curved retina cannot detect spatial resolution. Figure VI right part provide an explanation of how radially recessed photoreceptor cells within ommatidia contribute to spatial resolution detection.

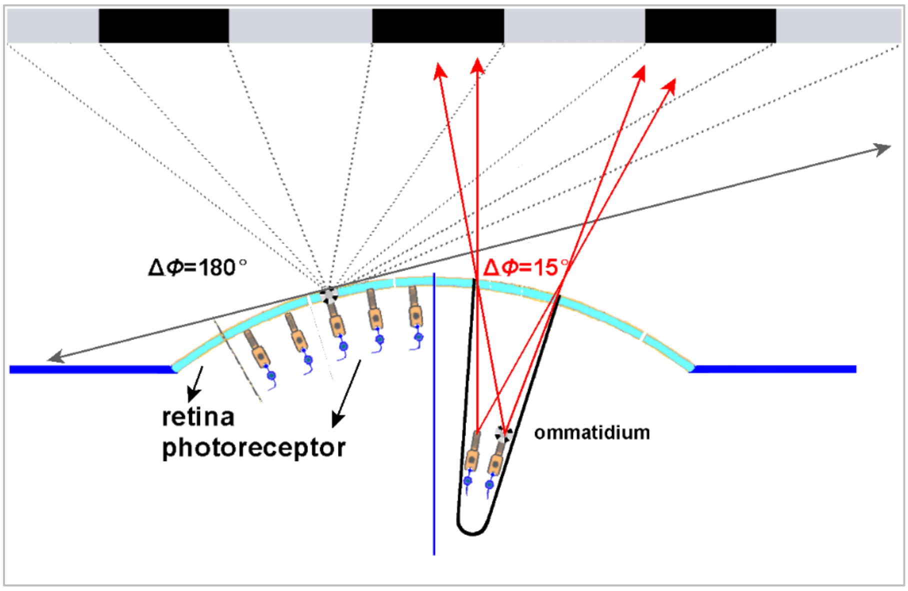

Fig. IX Field of photon reception for a photoreceptor on the outside of a convex retina surface. The third sensor from the left is representative for all photoreceptors in the retina. It has a field of view of 180 degrees and receiving direct light coming from the whole hemisphere. The two recessed photoreceptors in the ommatidium, drawn on the right, have a visual angle of 15 degrees

The third photoreceptor from the left has almost the same light activation level as all other shown sensors. The difference in light level between the photoreceptors is so small that detection of the grating of the white-black pattern in the background is not possible for the simple brain.

That becomes different when the photoreceptors are receding inwards to the curvature centre of the convex surface. The sides of the funnel are light tight. See example in Fig. IX. The field of view for the two photoreceptors is ~ 15 degrees and light comes from a small part of the background marked by the four arrows. More funnel shaped units together see different parts of the background and build a picture with a certain resolution of the background as a whole. Such a construction is found in the multi units' eyes of different animals. The 15 degrees is the resolution of the eyes of a deep-sea isopod [4].

Spatial resolution detection is possible with a concave curved retina. As can be seen in Fig. VIII, in phase 3 the light-sensitive sphere developed a concave retina in the posterior part of the sphere. The concave retina receives incident light only. In the retina as shown in Fig. VIII the inter-receptor angle between photoreceptor cell (c) and (b) is ~10 degrees. The eye sight is better than a deep-sea isopod but less then the eye sight of the cephalopod Nautilus [4].

Chosen principles for spatial resolution detection:

5. Spatial resolution detection is not possible on a convex photoreceptor surface.
6. Spatial resolution detection is possible in a convex curved surface with photoreceptors sunken in funnel-shaped ommatidia.
7. Spatial resolution detection in a one chamber eye needs a concave retina.

References

1. Albalat, R. (2012). Evolution of the genetic machinery of the visual cycle: a novelty of the vertebrate eye?. *Molecular biology and evolution*, *29*(5), 1461-1469. https://doi.org/10.1093/molbev/msr313
2. Jagger, W. S. (1992). The optics of the spherical fish lens. Vision Research, 32(7), 1271-1284. https://doi.org/10.1016/0042-6989(92)90222-5

3. Kusakabe, T. G., Takimoto, N., Jin, M., & Tsuda, M. (2009). Evolution and the origin of the visual retinoid cycle in vertebrates. Philosophical Transactions of the Royal Society B: Biological Sciences, 364(1531), 2897-2910. https://doi.org/10.1098/rstb.2009.0043
4. Land, M. F., & Nilsson, D. E. (2012)..: Animal eyes. OUP Oxford. ((2012). https://doi.org/10.1093/acprof:oso/9780199581139.001.0001)
5. Ramón Martínez-Morales, J., Rodrigo, I., & Bovolenta, P. (2004). Eye development: a view from the retina pigmented epithelium. *Bioessays*, *26*(7), 766-777. https://doi.org/10.1002/bies.20064
6. Nilsson, D.E.: The evolution of eyes and visually guided behaviour. Philosophical Transactions of the Royal Society B: Biological Sciences, 364(1531), 2833-2847 (2009). https://royalsocietypublishing.org/doi/10.1098/rstb.2009.0083
