# Supplementary material for: A possible origin of the inverted vertebrate retina revealed by physical modeling
Source: J Biol Phys. 2024 Aug 3;50(3-4):327–49. doi: 10.1007/s10867-024-09662-6 (PMC11490472; doi:10.1007/s10867-024-09662-6)
Supplement: Supplementary file 2 — Supplementary file2: Normalized local photoreceptor density (Eq. 1) where angles can be entered in degree. The presentation of Eq. 1 in degrees is given (PDF 97.9 KB) [file 10867_2024_9662_MOESM2_ESM.pdf]

A possible origin of the inverted vertebrate retina revealed by physical modeling

Journal of Biological Physics, Jan M.M. Oomens, independent researcher
oomens-science@ziggo.nl

Normalized local photoreceptor density (equation 1) where angles can be entered in degree.

In order to enter the angles in degrees, the equation 1 must be rewritten to correctly include the influence of the Sine function in the expression. The presentation of equation 1 in degrees is given in the blue box.

Normalized local photoreceptor density ; Box 1 ; equation 1

symbol list :

$\bar{\rho}_c$ = *mean photoreceptor density patch* ; ρ = *local photoreceptor density sphere*

R_c = *radius circular patch* ; R = *radius sphere* ; A_c = *patch area*

A = *surface area of the spherical cap* ; θ = *polar angle*; θ_{max} = *spanned angle*

normalized local photoreceptor density : $\dfrac{\rho}{\bar{\rho}_c} = \dfrac{R_c^2}{R^2} \cdot \dfrac{1}{\theta_{max}^2} \cdot \dfrac{\theta}{\sin(\theta)}$ *equation 1*

boundary condition $0 < \theta \le \theta_{max} < \pi$; θ *and* θ_{max} *in* (*rad*)

To transform this equation 1 into an expression with degree (°) as angle unit, special care has to be taken for the sin() function who has a numerical outcome.

$$1\text{rad} \equiv 57.296^\circ \;;\; \frac{\theta(rad)}{1rad} = \frac{\theta^\circ}{57.296^\circ} \;;\; \theta = \frac{1}{57.296^\circ}\cdot\theta^\circ \text{ and } \theta_{max} = \frac{1}{57.296^\circ}\cdot\theta^\circ_{max}$$

Equation 1 with angles θ° and θ°_{max} ; $\dfrac{\rho}{\bar{\rho}_c} = \dfrac{R_c^2}{R^2} \cdot \dfrac{1}{\left(\dfrac{1}{57.296^\circ}\cdot\theta^\circ_{max}\right)^2} \cdot \dfrac{\dfrac{1}{57.296^\circ}\cdot\theta^\circ}{\sin\left(\dfrac{1}{57.296^\circ}\cdot\theta^\circ\right)}$

$$\frac{\rho}{\bar{\rho}_c} = \frac{R_c^2}{R^2} \cdot \frac{57.296^\circ}{\left(\theta^\circ_{max}\right)^2} \cdot \frac{\theta^\circ}{\sin\left(\dfrac{\theta^\circ}{57.296^\circ}\right)}$$

; *boundary condition* $0^\circ < \theta^\circ \le \theta^\circ_{max} < 180^\circ$
