# Supplementary material for: A possible origin of the inverted vertebrate retina revealed by physical modeling
Source: J Biol Phys. 2024 Aug 3;50(3-4):327–49. doi: 10.1007/s10867-024-09662-6 (PMC11490472; doi:10.1007/s10867-024-09662-6)
Supplement: Supplementary file 3 — Supplementary file3: Normalized photoreceptor density Eqs. (2 and 3) where angles can be entered in degree (PDF 183 KB) [file 10867_2024_9662_MOESM3_ESM.pdf]

A possible origin of the inverted vertebrate retina revealed by physical modeling

Journal of Biological Physics, Jan M.M. Oomens, independent researcher

oomens-science@ziggo.nl

Normalized photoreceptor density equations (2&3) where angles can be entered in degree.

Equation 2 & 3, normalized local receptor density

angles θ and θ_{max} in degrees $(^\circ)$: *boundary condition* $0^\circ < \theta^\circ \le \theta^\circ_{max} < 180^\circ$

Equation 1

$$\frac{\rho}{\rho_c} = \frac{R_c^2}{R^2} \cdot \frac{1}{\left(\frac{\theta^\circ_{max}}{57.296^\circ}\right)^2} \cdot \frac{\frac{\theta^\circ}{57.296^\circ}}{\sin\left(\frac{\theta^\circ}{57.296^\circ}\right)} = \frac{R_c^2}{R^2} \cdot \frac{57.296^\circ}{(\theta^\circ_{max})^2} \cdot \frac{\theta^\circ}{\sin\left(\frac{\theta^\circ}{57.296^\circ}\right)}$$

Equation 2

$R = R_c$

$$\frac{\rho}{\rho_c} = \frac{57.296^\circ}{\theta^{\circ\,2}_{max}} \cdot \frac{\theta^\circ}{\sin\left(\frac{\theta^\circ}{57.296^\circ}\right)}$$

Equation 3

$$A = A_c \quad ; \quad \frac{R_c^2}{R^2} = 2 \cdot \left(1 - \cos\left(\frac{\theta^\circ_{max}}{57.296^\circ}\right)\right)$$

$$\frac{\rho}{\rho_c} = 2 \cdot \left(1 - \cos\left(\frac{\theta^\circ_{max}}{57.296^\circ}\right)\right) \cdot \frac{57.296^\circ}{(\theta^\circ_{max})^2} \cdot \frac{\theta^\circ}{\sin\left(\frac{\theta^\circ}{57.296^\circ}\right)}$$

Equation 2 & 3 expressed in degrees.

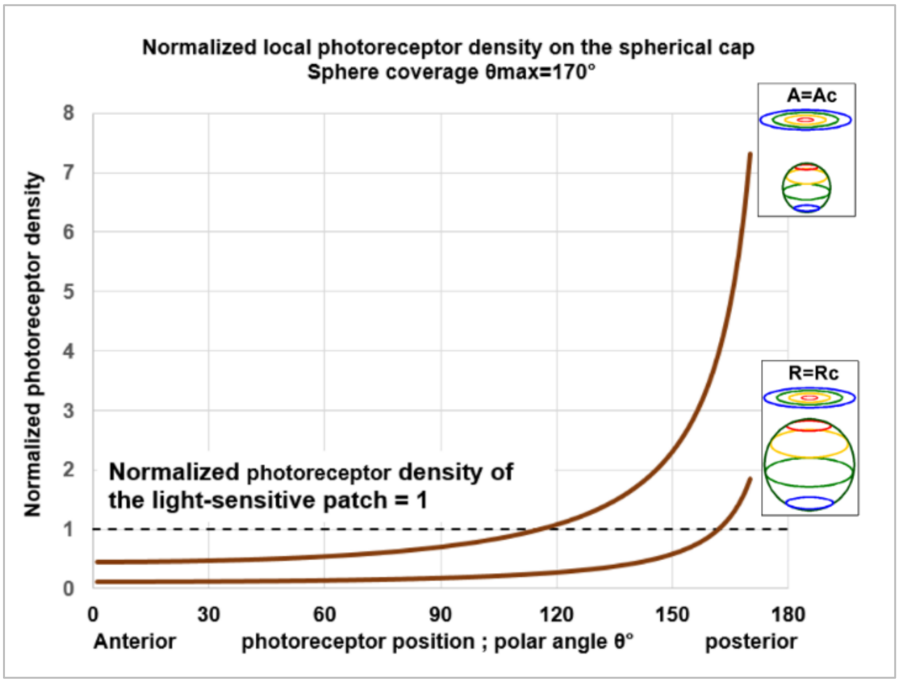

Equation 3 is used to calculate the constant surface area curve in figure 7. Top curve.

Equation 2 is used to calculate the constant radius curve in figure 7. Lower curve.
