# Supplementary material for: A possible origin of the inverted vertebrate retina revealed by physical modeling
Source: J Biol Phys. 2024 Aug 3;50(3-4):327–49. doi: 10.1007/s10867-024-09662-6 (PMC11490472; doi:10.1007/s10867-024-09662-6)
Supplement: Supplementary file 4 — Supplementary file4: Equations to transform photoreceptor position on the circular patch into the position on the lensball (PDF 270 KB) [file 10867_2024_9662_MOESM4_ESM.pdf]

A possible origin of the inverted vertebrate retina revealed by physical modeling

Journal of Biological Physics, Jan M.M. Oomens, independent researcher
oomens-science@ziggo.nl

Equations to transform photoreceptor position on the circular patch into the position on the lensball.

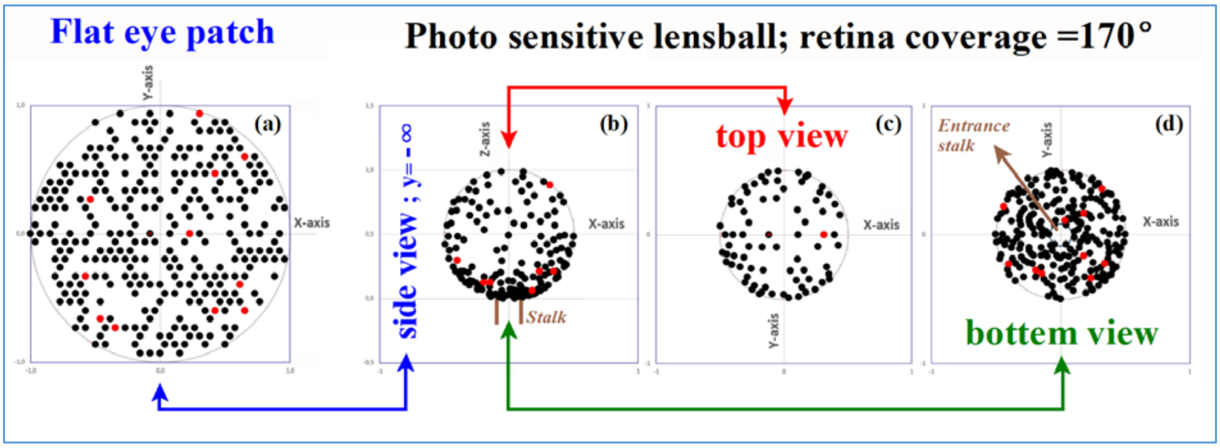

Figure 8. Computer simulation of how the 2D flat receptor cell configuration distributes spatially across the lensball. Figure 8 is created in EXCEL using the equations for the photoreceptor coordinates given on the next page.

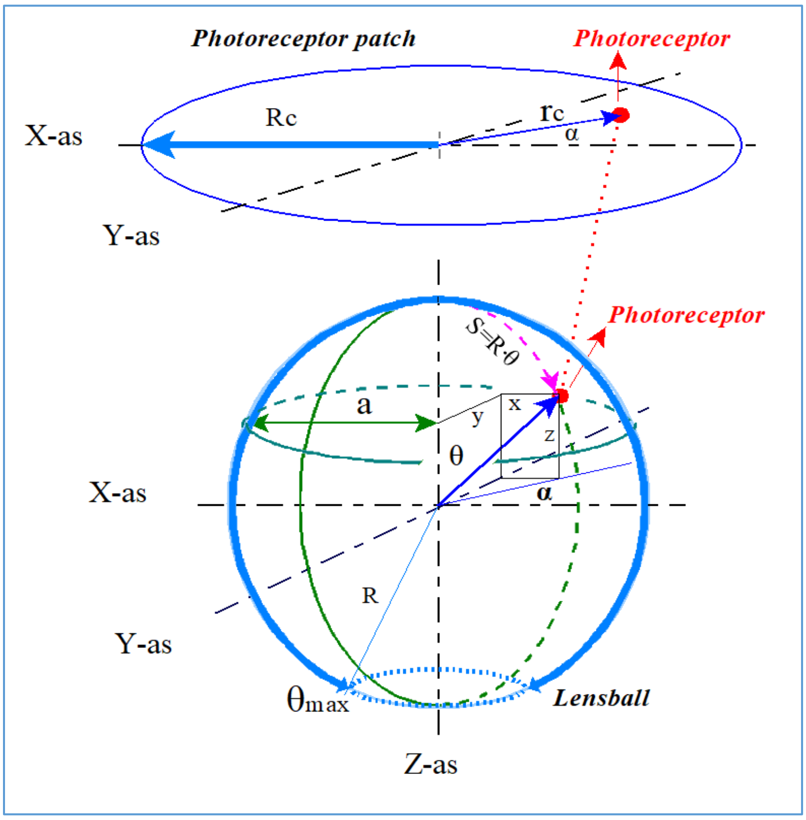

Individual sensor position calculation:

Rc=radius of the flat photoreceptor patch

R=radius lensball

Ac=surface area circular patch

A=surface area lensball covered with photoreceptors

S= R•θ; longitudinal arc length

θ_{max} = the lensball coverage angle of the surface area occupied by photoreceptors. It is marked by the fat blue curved line with an arrow on each end.

A possible origin of the inverted vertebrate retina revealed by physical modeling

Journal of Biological Physics, Jan M.M. Oomens, independent researcher
oomens-science@ziggo.nl

Photoreceptor position analysis

Surface area light sensitive circular patch $A_c = \pi \cdot R_c^{\,2}$

Choose any photoreceptor on the photoreceptor surface :

Photoreceptor position on patch::

$radius = r_c \quad ; \quad angle = \alpha$

$x_c = r_c \cdot \cos(\alpha) \quad ; \quad y_c = r_c \cdot \sin(\alpha)$

Assume a lensball coverage angle : $\theta_{max} = 170^\circ \equiv 2.967\, rad$

Surface area lensball cap $A = 2 \cdot \pi \cdot R^2 \cdot \int_0^{\theta_{max}} \sin\theta \cdot d\theta = 2 \cdot \pi \cdot R^2 - 2 \cdot \pi \cdot R^2 \cdot \cos(\theta_{max})$

For the condition $A = A_c$

$2 \cdot \pi \cdot R^2 - 2 \cdot \pi \cdot R^2 \cdot \cos\theta_{max} = \pi \cdot R_c^{\,2} \quad ; \quad R^2 \cdot (2 - 2 \cdot \cos(\theta_{max})) = R_c^{\,2}$

Lensball cap radius: $R = \sqrt{\dfrac{R_c^{\,2}}{2 \cdot (1 - \cos(\theta_{max}))}}$

Longitudinal stretch factor : $\sigma = \dfrac{R \cdot \theta_{max}}{R_c} = \theta_{max} \cdot \sqrt{\dfrac{1}{2 \cdot (1 - \cos(\theta_{max}))}}$

Photoreceptor position on ball cap:

Longitudinal arc length from pole to photoreceptor: $S = R \cdot \theta = r_c \cdot \sigma$

Longitudinal angle from pole to photoreceptor: $\theta = \dfrac{S}{R} = \dfrac{r_c}{R} \cdot \sigma$

The distance from the lensball z-axis: $a = R \cdot \sin(\theta)$

Coordinates of the photoreceptor on the lensball:

$x = a \cdot \cos(\alpha) = R \cdot \sin(\theta) \cdot \cos(\alpha)$

$y = a \cdot \sin(\alpha) = R \cdot \sin(\theta) \cdot \sin(\alpha)$

$z = R \cdot \cos(\theta)$
