# Supplementary material for: A possible origin of the inverted vertebrate retina revealed by physical modeling
Source: J Biol Phys. 2024 Aug 3;50(3-4):327–49. doi: 10.1007/s10867-024-09662-6 (PMC11490472; doi:10.1007/s10867-024-09662-6)
Supplement: Supplementary file 5 — Supplementary file5: Eq. 4 is used to examine the image structure on the retina and to calculate the maximum blur spot size (PDF 257 KB) [file 10867_2024_9662_MOESM5_ESM.pdf]

A possible origin of the inverted vertebrate retina revealed by physical modeling

Journal of Biological Physics, Jan M.M. Oomens, independent researcher
oomens-science@ziggo.nl

Equation 4 is used to examine the image structure on the retina and to calculate the maximum blur spot size.

Derivation of this equation 4 is given in two different ways:

a) using standard and inverse trigonometric functions
b) using standard trigonometric functions.

Equation 4, Normalised radius of photon contact point

$$\frac{r}{R} = \sin\left(2 \cdot \arcsin\left(\frac{n_1}{n_2} \cdot \sin\beta\right) - \theta\right) \quad \textit{equation 4}$$

β *and* θ *in* (rad)

symbol list:

β = angle of incident
ε = polar angle contact point
γ = angle of refraction
θ = polar angle incident point
n_1 = refractive index medium
n_2 = refractive index lensball
r = radius photon contact point
R = radius lensball

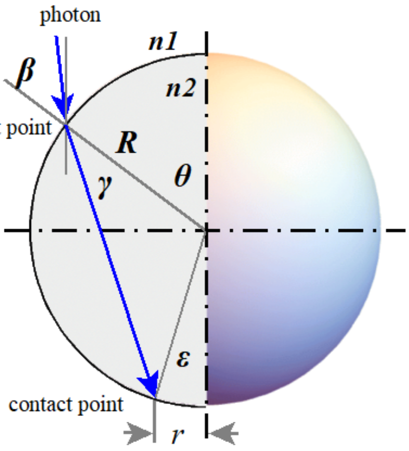

(a) Derivation of equation 4 using standard and inverse trigonometric functions

Equation 4 using inverse trigonometric functions allows the shortest equation notation.

Blur spot , derivation of the radius of the photon contact point on the retina

$$\varepsilon + \theta + (180^\circ - 2 \cdot \gamma) = 180^\circ \quad ; \quad \varepsilon = 2 \cdot \gamma - \theta$$

Snellius law ; $n_1 \cdot \sin\beta = n_2 \cdot \sin\gamma$

$$\sin\gamma = \frac{n_1}{n_2} \cdot \sin\beta \quad ; \quad \gamma = \arcsin\left(\frac{n_1}{n_2} \cdot \sin\beta\right)$$

Blur spot radius : $r = R \cdot \sin\varepsilon = R \cdot \sin(2 \cdot \gamma - \theta)$

Normalised blur spot radius ; $\frac{r}{R} = \sin(2 \cdot \gamma - \theta)$

$\frac{r}{R}$ expressed in β and θ

$$\frac{r}{R} = \sin\left(2 \cdot \arcsin\left(\frac{n_1}{n_2} \cdot \sin\beta\right) - \theta\right)$$

A possible origin of the inverted vertebrate retina revealed by physical modeling
Journal of Biological Physics, Jan M.M. Oomens, independent researcher
oomens-science@ziggo.nl

(b) Derivation of equation 4 using standard trigonometric functions

Derivation of equations to calculate the normalised photon contact point radius

The relation between the measures of the angles $\varepsilon, \theta, \gamma$ in radians is:

$\varepsilon + \theta + (\pi - 2 \cdot \gamma) = \pi \quad ; \quad \varepsilon = 2 \cdot \gamma - \theta$

Normalised photon contact point radius equals $\frac{r}{R} = \sin(\varepsilon) = \sin(2 \cdot \gamma - \theta)$

TrigExpand (Wolfram Mathematica) is used to express $\frac{r}{R}$ in standard trigonometric functions

$$\frac{r}{R} = 2 \cdot \cos\theta \cdot \sin\gamma \cdot \cos\gamma + \sin\theta \cdot \sin\gamma^2 - \sin\theta \cdot \cos\gamma^2$$

Snellius law; $(n_1 \cdot \sin\beta = n_2 \cdot \sin\gamma)$ provides the relation between angles (β) and (γ)

$$\sin\gamma = \frac{n_1}{n_2} \cdot \sin\beta \quad ; \quad \cos\gamma^2 = 1 - \sin\gamma^2 \quad ; \quad \cos\gamma = \sqrt{\left(1 - \left(\frac{n_1}{n_2}\right)^2 \cdot \sin\beta^2\right)}$$

$\frac{r}{R}$ expressed in angles β and θ and trigonometric function sin()

$$\frac{r}{R} = 2 \cdot \sqrt{1 - \sin\theta^2} \cdot \frac{n_1}{n_2} \cdot \sin\beta \cdot \sqrt{1 - \left(\frac{n_1}{n_2}\right)^2 \cdot \sin\beta^2} + \sin\theta \cdot \left(\frac{n_1}{n_2}\right)^2 \cdot \sin\beta^2 - \sin\theta \cdot \left(1 - \left(\frac{n_1}{n_2}\right)^2 \cdot \sin\beta^2\right)$$

When $\beta = \theta$ the equation for the normalised photon contact point radius $\frac{r}{R}$ can be simplified

$$\frac{r}{R} = 2 \cdot \sqrt{1 - \sin\theta^2} \cdot \frac{n_1}{n_2} \cdot \sin\theta \cdot \sqrt{1 - \left(\frac{n_1}{n_2}\right)^2 \cdot \sin\theta^2} + \sin\theta \cdot \left(\frac{n_1}{n_2}\right)^2 \cdot \sin\theta^2 - \sin\theta \cdot \left(1 - \left(\frac{n_1}{n_2}\right)^2 \cdot \sin\theta^2\right)$$

$$\frac{r}{R} = \sin\theta \cdot \left(2 \cdot \frac{n_1}{n_2} \cdot \left(\sqrt{(1 - \sin\theta^2) \cdot \left(1 - \left(\frac{n_1}{n_2}\right)^2 \cdot \sin\theta^2\right)} + \left(\frac{n_1}{n_2}\right) \cdot \sin\theta^2\right) - 1\right)$$
