# Supplementary material for: A possible origin of the inverted vertebrate retina revealed by physical modeling
Source: J Biol Phys. 2024 Aug 3;50(3-4):327–49. doi: 10.1007/s10867-024-09662-6 (PMC11490472; doi:10.1007/s10867-024-09662-6)
Supplement: Supplementary file 6 — Supplementary file6: Derivation of the modified cup depth to analyse the influence of a positive lens in the aperture of a retina cup on the maximum detectable spatial resolution (PDF 214 KB) [file 10867_2024_9662_MOESM6_ESM.pdf]

A possible origin of the inverted vertebrate retina revealed by physical modeling

Journal of Biological Physics, Jan M.M. Oomens, independent researcher
oomens-science@ziggo.nl

Derivation of the modified cup depth to analyse the influence of a positive lens in the aperture of a retina cup on the maximum detectable spatial resolution.

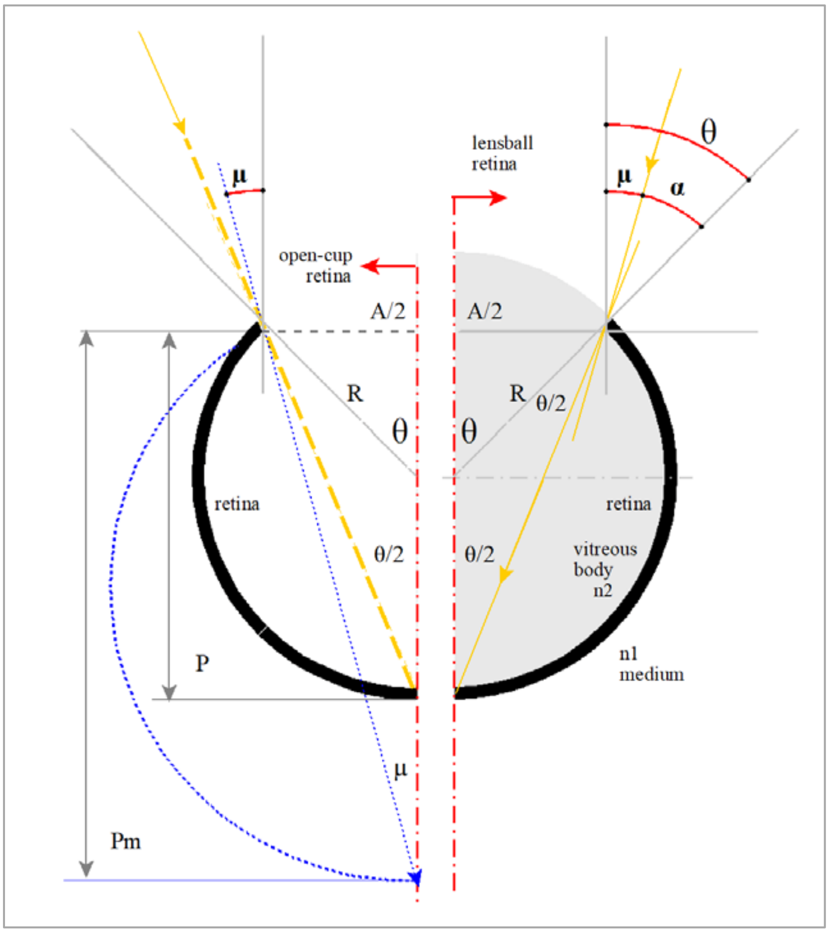

The figure presents the cross-sections of two types of retina cup.

The left side is half the cross-section of an open-cup retina.

On the right is half of the cross-section of a lensball retina.

The radius (symbol: R) and the aperture diameter (symbol: A) is the same size for both retina cups.

The solid angle (symbol: θ/2) is a measure of the amount of the field of view from the posterior pole of the open-cup retina.

The field of view from the posterior pole of the open-cup retina, with solid angle θ/2, is bigger than the field of view from the posterior pole of the lensball retina cup, with solid angle μ, caused by refraction on the lensball ($n2 > n1$ then $\theta/2 > \mu$).

The smaller field of view from the posterior pole of the lensball retina cup means a higher detectable spatial resolution.

Symbol list:

- α = The angle to the normal at which the photon must strike the incident point to hit the posterior pole
- θ = Polar angle of aperture rim
- μ = The angle between the optical axis and the trajectory of the incoming photon at angle α
- n_1 = Refractive index medium
- n_2 = Refractive index lensball
- P = Posterior nodal distance or cup depth
- P_m = Modified posterior nodal distance
- R = Radius of the retina cup
- $A/2$ = Aperture radius of the retina cup

A possible origin of the inverted vertebrate retina revealed by physical modeling
Journal of Biological Physics, Jan M.M. Oomens, independent researcher
oomens-science@ziggo.nl

Derivation of the modified pit depth P_m to account for lens influence

Open pit retina cup: *pit depth* $P = R\cdot(1+\cos\theta)$

Modified pit depth for the lensball retina cup:

$\theta = \mu + \alpha$; The relation between $\frac{\theta}{2}$ and α is given

by *Snellius law*: $\left(n_1\cdot\sin\alpha = n_2\cdot\sin\left(\frac{\theta}{2}\right)\right)$; $\sin\alpha = \frac{n_2}{n_1}\cdot\sin\left(\frac{\theta}{2}\right)$

$\alpha = \arcsin\left(\frac{n_2}{n_1}\cdot\sin\left(\frac{\theta}{2}\right)\right)$; $0 \le \theta \ge \frac{\pi}{2}$

The distance from the aperture to the posterior nodal point in the modified open retina cup is the modified pit depth P_m.

$$\tan\mu = \frac{A/2}{P_m} = \frac{R\cdot\sin\theta}{P_m} \;;\; P_m = R\cdot\frac{\sin\theta}{\tan\mu} = R\cdot\frac{\sin\theta}{\tan(\theta-\alpha)}$$

$$P_m = \frac{R\cdot\sin\theta}{\tan\left(\theta - \arcsin\left(\frac{n_2}{n_1}\cdot\sin\left(\frac{\theta}{2}\right)\right)\right)} \qquad \textit{equation } 6$$

boundary condition: $\frac{n_2}{n_1}\cdot\sin\left(\frac{\theta}{2}\right) \in [-1,1]$; θ *in* (*rad*)

The field of view from the posterior pole of the lensball retina cup, with solid angle µ, can be simulated in the open-cup retina by a modified cup size with a posterior nodal distance P_m (n2 > n1 then P_m > P).

Equation 6, modified aperture depth

The influence on the maximum detectable spatial frequency of a positive lens in the aperture in a cup shaped retina can be calculated by a parameter P_m representing a modified posterior nodal distance P .

Supersede parameter P in eqn 5 by P_m

$$\nu_{max} = 0.375\cdot\left(\frac{P}{A}\right)\cdot\sqrt{\ln\left(0.746\cdot A^2\cdot\sqrt{I}\right)} \qquad \textit{equation } 5$$

(Nilsson & Pelger 1994) to calculate the maximum detectable spatial frequency in a cup shaped retina with a positive lens in the aperture.

symbol list:
ν_{max} = maximum detectable spatial frequency
A = diameter aperture
P = posterior nodal distance or cup depth
I = photons/nodal distance

The maximum spatial resolution of the lensball retina cup can be calculated by superseding P_m (equation 6) in equation 5 (Nilsson & Pelger 1994) while keeping the light entry opening constant.
